# Secretome of Cancer-Associated Fibroblasts (CAFs) Influences Drug Sensitivity in Cancer Cells

**DOI:** 10.1021/acs.jproteome.4c00112

**Published:** 2024-05-20

**Authors:** Rachel Lau, Lu Yu, Theodoros I. Roumeliotis, Adam Stewart, Lisa Pickard, Jyoti S. Choudhary, Udai Banerji

**Affiliations:** † Clinical Pharmacology and Adaptive Therapy Group, The Institute of Cancer Research and The Royal Marsden NHS Foundation Trust, London SM2 5PT, United Kingdom; ‡ Functional Proteomics Group, Chester Beatty Laboratories, The Institute of Cancer Research, London SW3 6JB, United Kingdom

**Keywords:** Stroma, secretome, cancer, drug resistance

## Abstract

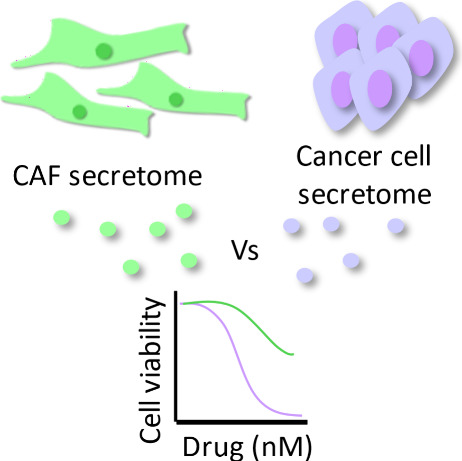

Resistance is a major problem with effective cancer treatment and the stroma forms a significant portion of the tumor mass but traditional drug screens involve cancer cells alone. Cancer-associated fibroblasts (CAFs) are a major tumor stroma component and its secreted proteins may influence the function of cancer cells. The majority of secretome studies compare different cancer or CAF cell lines exclusively. Here, we present the direct characterization of the secreted protein profiles between CAFs and *KRAS* mutant-cancer cell lines from colorectal, lung, and pancreatic tissues using multiplexed mass spectrometry. 2573 secreted proteins were annotated, and differential analysis highlighted understudied CAF-enriched secreted proteins, including Wnt family member 5B (WNT5B), in addition to established CAF markers, such as collagens. The functional role of CAF secreted proteins was explored by assessing its effect on the response to 97 anticancer drugs since stromal cells may cause a differing cancer drug response, which may be missed on routine drug screening using cancer cells alone. CAF secreted proteins caused specific effects on each of the cancer cell lines, which highlights the complexity and challenges in cancer treatment and so the importance to consider stromal elements.

## Introduction

The tumor microenvironment is a key contributing factor to cancer progression and drug response,^1,2^ particularly as it is a major source of secreted proteins which form the secretome. Profiling proteins secreted from a cell population highlights potential drivers that are likely to activate or suppress signaling pathways between cells and can be indicative of prognosis and response to therapy.

The tumor microenvironment is complex with various stromal cell types where cancer-associated fibroblasts (CAFs) are one of the most abundant.^3,4^ Therefore, we focused on CAFs to break down the contributions of individual stromal cells. Furthermore, we focused on a group of *KRAS* mutant-cancer cell lines. Cancers driven by *KRAS* mutations, such as nonsmall cell lung cancer (NSCLC), pancreatic adenocarcinoma (PDAC), and colorectal cancer (CRC), are common and associated with poor outcomes and are an area of unmet need.^5^ How cancer-associated fibroblasts (CAFs) affect drug response in *KRAS* mutant-cancer cells has not been well explored.

While there are multiple publications related to the secretome of cancer cells^6−8^ and CAFs,^9,10^ we believe this is the first study to profile and directly compare the secretome of CAFs and cancer cell lines in a multiplexed manner and to functionally assess the differentially expressed secreted proteins by undertaking a bespoke drug screen of 97 anticancer drugs.

## Experimental Procedures

### Cell Culture

The following cell lines were used: Colorectal and lung CAFs (VitroBiopharma, lots 001A and 002A), H747 and H2030 (ATCC); LIM2099 (PHE); SW620 (Sigma); H1792 and H23 (donated by Prof. Julian Downward); CAPAN1, DANG, and MIAPACA2 (donated by Dr. Anguraj Sadanandam); and PSCs (ScienCell, lot 14289). All cell lines were cultured in a 5% CO_2_, 37 °C incubator with a humidified atmosphere. All cell lines have been authenticated by ATCC short tandem repeat profiling and were routinely checked for mycoplasma. All cell lines were grown in Dulbecco’s Modified Eagle Medium/Nutrient Mixture F-12 media (ThermoFisher Scientific) supplemented with 10% fetal bovine serum (FBS; ThermoFisher Scientific, lot 2079409), 2 mM l-glutamine (ThermoFisher Scientific), and 1% nonessential amino acids (Sigma).

### Sample Preparation for Secretome Analysis

**Figure 1:**
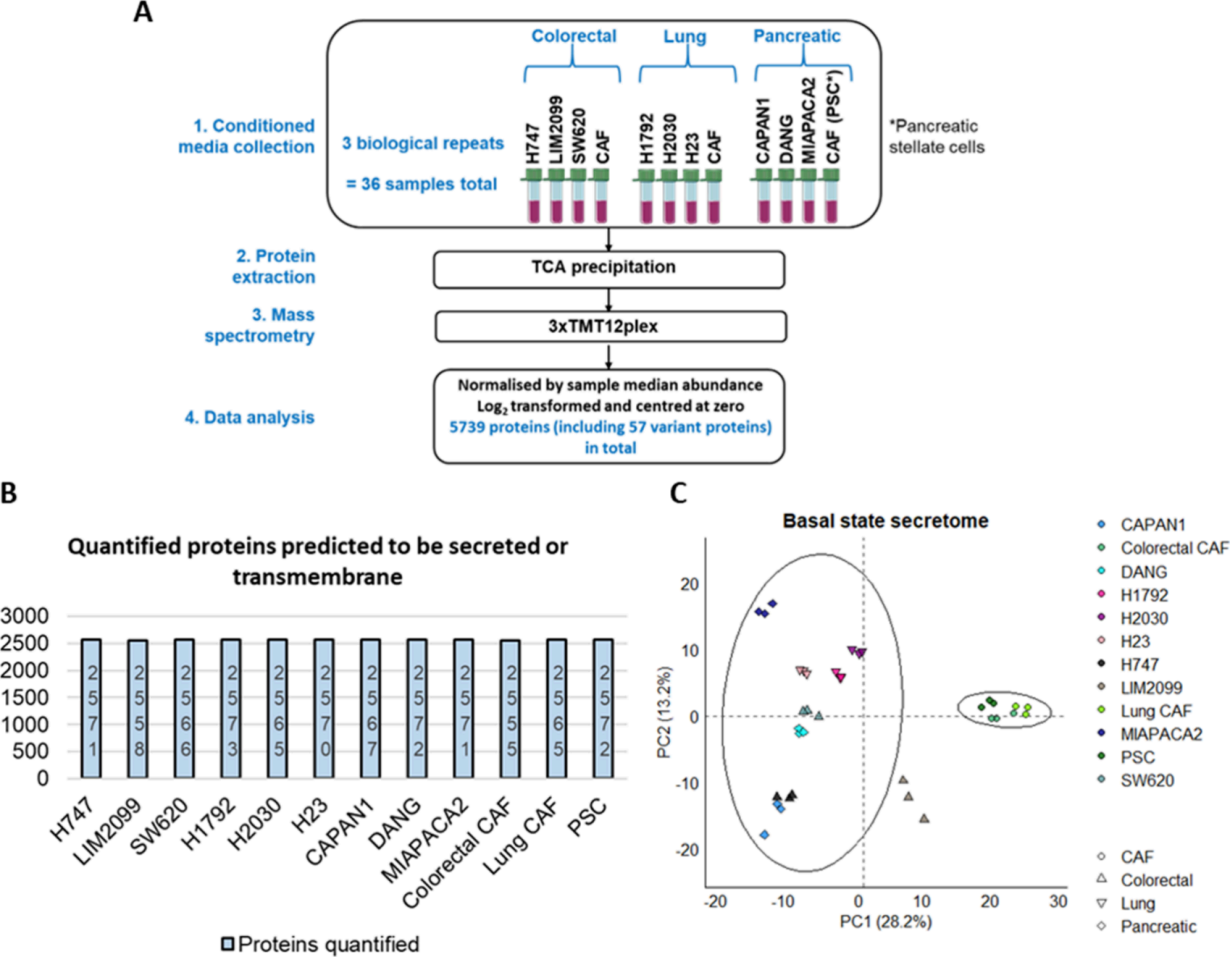
Basal state secretome analysis. (A) The basal state secretome was obtained from conditioned media samples derived from 12 cell lines (three cancer-associated fibroblast (CAF) cell line models, three *KRAS* mutant-colorectal cancer, three *KRAS* mutant-lung cancer, and three *KRAS* mutant-pancreatic cancer cell lines) in a 12plex with each run representing biological replicates. Three tandem mass tag (TMT) batches were run. Protein was extracted using trichloroacetic acid (TCA) precipitation. Protein abundance was normalized by sample median abundance, log_2_ transformed, and centered at zero. The tube images are from Servier Medical Art, licenced under a Creative Commons Attribution 3.0 unported licence. (B) Barplot of the proteins predicted to be secreted or transmembrane in the basal state secretome of the 12 cell lines. (C) Principal component analysis of the proteins annotated to be secreted or transmembrane in the basal state secretome analysis of the 12 cell lines. PSC = pancreatic stellate cells.

The sample preparation for secretome analysis is summarized in Figure 1A. Conditioned media (CM) were prepared by culturing the cells to approximately 60% confluence and washing the cells with phosphate buffered saline (PBS, ThermoFisher Scientific) and serum free media and adding 20 mL of serum free media for 24 h of incubation. Twenty-four hours was used to generate CM for mass spectrometry analysis to minimize the time the cells are serum deprived but enough time to obtain a snapshot of the proteins secreted in proliferating cells. Upon harvesting the CM, the cell viability was checked using trypan blue where all samples had >85% viability after serum deprivation. The CM was centrifuged briefly to remove any cellular debris, filtered using a 0.2 μm filter and stored at −80 °C for downstream processing. Proteins in the CM were reduced by 5 mM tris(2-carboxyethyl)phosphine (TCEP) (Sigma) at 56 °C for 30 min and alkylated by 10 mM iodoacetamide (IAA) (Sigma) for 30 min at room temperature. 20% trichloroacetic acid (TCA) was added and left on ice for 1 h and centrifuged at 21,000*g* for 10 min. The protein pellet was resuspended in 100 mM triethylammonium bicarbonate buffer (TEAB) and 2 μg trypsin (Pierce, MS-Grade) was added to digest the proteins at 37 °C for 18 h with shaking. The digested samples were dried in SpeedVac completely, redissolved in ACN/H_2_O mixture, and SpeedVac dried completely again. The dried samples were redissolved in water, and peptide concentrations were measured by nanodrop at A280 nm. 20 μg of peptides per sample was taken for TMTpro 16plex (ThermoFisher Scientific) labeling. The samples were labeled as H1792 (128C), H2030 (129N), H23 (129C), CAPAN1 (130N), DANG (130C), MIAPACA2 (131N), H747 (131C), LIM2099 (132N), SW620 (132C), Lung CAF (133N), PSC (133C), and Colorectal CAF (134N). The samples were resuspended in 0.1% NH_4_OH/100% H_2_O and fractionated on an XBridge BEH C18 column (2.1 mm i.d. × 150 mm, Waters) with an initial 5 min loading then a linear gradient from 5% ACN/0.1% NH_4_OH (pH 10) to 35% CH_3_CN/0.1% NH_4_OH in 30 min, then to 80% CH_3_CN/0.1% NH_4_OH in 5 min and stayed for another 5 min. The flow rate was 200 μL/min. Fractions were collected at every 42 s from retention time from 7.8 min to 50 min and then concatenated to six fractions and dried in a SpeedVac. Samples were then resuspended in 0.5% formic acid (FA) for LC-MS/MS analysis.

### Mass Spectrometry Analysis

25% of the peptides were injected. The LC-MS/MS analysis was performed on the Orbitrap Fusion Lumos mass spectrometer coupled with a U3000 RSLCnano UHPLC system. All instruments and columns used are from ThermoFisher Scientific. Peptides were first loaded on a PepMap C18 nanotrap (100 μm i.d. × 20 mm, 100 Å, 5 μm) at 10 μL/min with 0.1% FA/H2O and then separated on a PepMap C18 column (75 μm i.d. × 500 mm, 100 Å, 2 μm) at 300 nL/min with a linear gradient of 8–32% ACN/0.1% FA. The LC-MS/MS run was 180 min with the LC gradient for 150 min. The data acquisition used standard data-dependent acquisition mode with a cycle time at 3 s. The MS1 survey scan was between *m*/*z* 375 and 1500 at 120 000 resolution and the automatic gain control (AGC) at 100 000 with maximum injection time at 50 ms. The MS acquisition on multiply charged ions (+2 to +6) with an intensity above 10 000 was fragmented in high energy collisional dissociation (HCD) at 36% normalized collision energy (NCE), with an isolation width at 0.7 Da in a quadrupole and detected in Orbitrap in scan mode of the defined first *m*/*z* at 100. The resolution was set at 50 000 at *m*/*z* 200 and the AGC at 100 000 with a maximum injection time at 86 ms. The dynamic exclusion was set at 45 s with ±7 ppm mass tolerance.

### Mass Spectrometry Data Processing

All raw files were processed in Proteome Discoverer 2.4 (ThermoFisher Scientific) using Sequest HT to search against a reviewed *Homo sapiens* Uniprot database (March 2021), cell line specific variant databases from Cosmic (April 2021), and contaminate database (ThermoFisher Scientific).

Search parameters were as follows: trypsin with two maximum miss-cleavage sites, mass tolerances at 20 ppm for the precursor, and 0.1 Da for the fragment ions; deamidation (N, Q) and oxidation (M) as dynamic modification; and carbamidomethyl (C) and TMTpro (K, N-terminus) as static modification. Peptides were validated by Percolator with the *q*-value set at 0.01 for the decoy database search, and only highly confident PSMs (Peptide Spectrum Matches) were considered. Only master proteins were reported. For reporter ion intensity detection, the reporter ion quantifier node parameters were an integration window tolerance of 20 ppm and an integration most-confident centroid for peak detection at the MS2 level. Only unique peptides were considered for quantification. The TMTpro Quan value correction factor, provided by the manufacturer’s certificate of analysis, was applied. Reported ion intensities were normalized by total peptide amount and then scaled on the average to correct the variation by different protein loadings in each channel.

The protein abundance was corrected for equal loading across samples by median normalization for each sample, log_2_ transformation, and centering around zero by subtracting the mean protein abundance according to TMT batch.

### Drug Screen Analysis

CM was prepared fresh after 48 h incubations in the appropriate cell lines, and the harvested media were used to seed cancer cells onto 384 well plates. For the initial drug screen, 24 h after seeding the cells with the appropriate CM, the cells were treated with a custom Apexbio library of 97 different drugs (Supplementary Table 1) using the Echo acoustic liquid dispenser 550 (Labcyte) at concentrations of 0.06, 0.3, 0.6, 2, 5, and 10 μM (1% (v/v) DMSO final) with no technical repeats. 72 h after treatment, cell viability was measured using CellTiter-Blue assays (Promega). The percent inhibition was calculated on Dotmatics where the average standard *Z* prime of the plates in the drug screen was 0.54.

Any drugs where the percent inhibition exceeded 55 for both cancer and CAF CM were assessed again at lower concentrations of 50, 20, 10, 3, 1.5, and 0.3 nM with no technical repeats. On the other hand, any drugs where the percent inhibition was below 45% for both cancer and CAF CM were assessed again at higher concentrations of 0.6, 3, 6, 20, 50, and 100 μM with no technical repeats.

At each concentration per cancer cell line, the drug hits were identified using Vortex (Dotmatics) if the difference between the cancer and CAF-derived CM drug responses (delta) was more than 2 standard deviations away from the mean delta of all of the drugs at that specific concentration in the cancer cell line.

The drug hits were then validated using an 11 point IC_50_ curve (0.5% (v/v) DMSO final) using a new batch of the drugs in three independent experiments (each with three technical repeats) using an Echo acoustic liquid dispenser 550 (Labcyte), and cell viability was measured using CellTiter-Blue assays (Promega) 72 h after treatment. Four parameter logistic IC_50_ curves were generated using Graphpad (version 8).

### Statistics and Bioinformatics

All analyses and plots were generated using R version 4.2. The *t* test was undertaken using the matrixTests package (v0.1.9.1) with Storey’s *q* value calculated using the qvalue package (v2.30.0). Functional enrichment analysis was undertaken using the EnrichR package (v3.2) using the “Gene Ontology Biological Process 2021” database.

Classically secreted proteins were predicted using SignalP, version 5.^11^ Nonclassically secreted proteins were predicted using SecretomeP, version 2.^12^ Transmembrane helices in proteins were predicted using TMHMM server version 2,^13^ and other transmembrane proteins were mined for using Cell Surface Protein Atlas (CSPA) validated entries^14^ and Surfaceome.^15^ Other secreted ligands were datamined using FANTOM5.^16^ Microvesicle proteins were datamined using Vesiclepedia.^17^

## Results

### Characterizing the Differential Secreted Proteins between CAFs and KRAS Mutant-Cancer Cells

The secretome can be characterized using media extracted from cell culture, also known as conditioned media (CM). Mass spectrometry analysis allows for an unbiased global search for the secreted proteins, but a typical cell culture medium has a wide dynamic range with the lowly abundant secreted proteins that can be as low as nanograms per milliliter among the highly abundant serum components, which are often in the milligrams per milliliter range. Serum deprivation is a conventional and simple method to reduce the dynamic range of the CM samples, and so CM samples represent a starting point in discovery research on CAF-enriched secreted proteins.

The basal state secretome was characterized using CM samples from three CAF cell line models (colorectal CAF, lung CAF, and pancreatic stellate cells (PSC)), three *KRAS* mutant-CRC cells (H747, LIM2099, SW620), three *KRAS* mutant-NSCLC cells (H1792, H2030, H23), and three *KRAS* mutant-PDAC cells (CAPAN1, DANG, MIAPACA2) in a multiplexed manner (Figure 1A). This was undertaken in triplicates, each representing biological repeats. Therefore, 36 CM samples were analyzed in total, which quantified 5739 proteins (including 57 variant proteins). The cell viability was >85% upon harvesting the CM after serum deprivation (Supplementary Figure 1A), so the proteins captured are from viable cells.

Proteins can be secreted into the extracellular space through classical or nonclassical secretory pathways.^18,19^ Classical secretion occurs via the endoplasmic reticulum (ER)/Golgi while nonclassical secretion of proteins that lack a signal peptide can occur through various pathways, including by direct translocation, via an ABC transporter, through membrane-bound organelles or bypassing the Golgi. Furthermore, transmembrane proteins can exist on microvesicles or can be cleaved off and released into the extracellular space.

Therefore, secreted or transmembrane proteins were identified using different software and databases (SignalP,^11^ SecretomeP,^12^ TMHMM,^13^ Cell Surface Protein Atlas (CSPA),^14^ surfaceome,^15^ FANTOM5,^16^ and Vesiclepedia^17^). Of the 5739 proteins quantified, 2573 proteins (including 22 variant proteins) were annotated to be secreted or transmembrane (Table 1 and Supplementary Table 2) where over 2550 proteins were quantified across all cell lines (Figure 1B). There is minimal variation between the biological repeats for secretome analysis, and PCA highlighted a distinct separation between cancer and CAF cell line models (Figure 1C).

**Table 1:** Number of Proteins Predicted to Be Secreted or Transmembrane in the Basal State Secretome Analysis^a^

	number of proteins
total proteins identified by mass spectrometry	5739 (57 variants)
SignalP (classical secretion)	977 (11 variant)
SecretomeP (nonclassical secretion)	1004 (3 variant)
TMHMM (transmembrane proteins)	808 (6 variant)
Cell surface protein atlas (transmembrane proteins)	672 (8 variant)
Surfaceome (transmembrane proteins)	458 (3 variant)
FANTOM5 (signaling ligands)	263 (6 variant)
Vesiclepedia (extracellular vesicle)	460 (5 variant)
total proteins predicted to be secreted^b^	2573 (22 variant)

a Proteins were predicted to be secreted or transmembrane using different prediction software (SignalP, SecretomeP, TMHMM) and databases (Cell Surface Protein Atlas, FANTOM5, surfaceome, and Vesiclepedia).

b Note that some proteins were identified in multiple prediction software and databases.

We previously reported the basal state proteome profiles of our cell lines of interest.^20^ Of the secreted or transmembrane proteins identified in our secretome analysis that were also detected in our proteome analysis, there was a high correlation in the protein abundance between the two data sets (Supplementary Figure 1B). This indicates that our secretome analysis is reflective of viable cells, given that our basal sate proteome analysis involved cells cultured under the normal 10% serum conditions.

**Figure 2:**
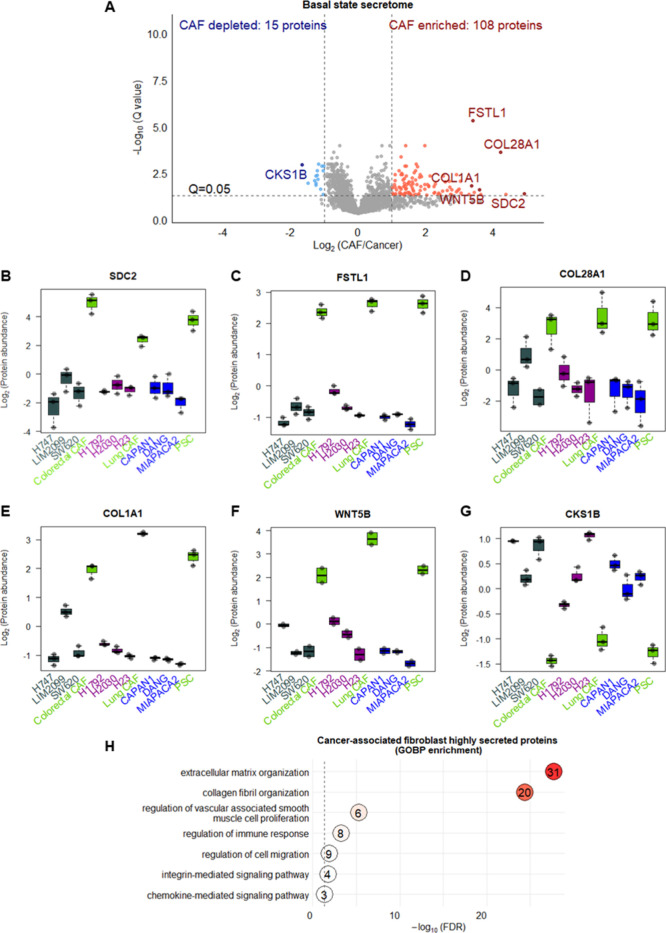
Differentially expressed secreted proteins between cancer-associated fibroblasts (CAFs) and cancer cell lines. (A) Volcano plot of the *t* test analysis on the annotated secreted and transmembrane proteins between the cell types (CAFs vs cancer), whereby significant differentially expressed proteins were defined by the cutoffs 2-fold (abs.log2 > 1) and Storey’s *Q* value < 0.05 (−log10(0.05) = 1.3). Boxplot of the protein expression of (B) syndecan 2 (SDC2), (C) follistatin like 1 (FSTL1), (D) collagen type 28 alpha 1 (COL28A1), (E) collagen type I alpha 1 (COL1A1), (F) wnt family member 5B (WNT5B), and (G) cyclin-dependent kinase regulatory subunit (CKS1B). (H) Gene Ontology Biological Process (GOBP) enrichment analysis of the CAF highly secreted proteins where the significance of the enrichment and the number of significant differentially expressed proteins within each GOBP term are detailed. PSC = pancreatic stellate cells. Green = CAF, gray = colorectal cancer, purple = lung cancer, blue = pancreatic cancer.

To determine the differentially expressed secreted or transmembrane proteins between CAFs and *KRAS* mutant-cancer cells, the protein abundance was compared between the cell lines grouped by cell types: CAFs or cancer (Figure 2A). 123 proteins (including 2 variant proteins) were found to have >2-fold significant differential expression (abs.log_2_ > 1, *Q* < 0.05) between the two cell types where the majority were enriched in the CAF CM (108 proteins where two of which are variant) while 15 proteins had higher levels in the *KRAS* mutant-cancer CM (Supplementary Table 3).

Of the proteins with the highest fold difference, some have been reported to be upregulated in CAFs, such as syndecan 2 (SDC2),^21^ follistatin like 1 (FSTL1),^22^ and collagens,^23,24^ including COL28A1 and COL1A1 (Figure 2B–F). Notably, Wnt family member 5B (WNT5B) was also identified as one of the highest CAF-enriched secreted proteins (Figure 2G), but there are minimal studies on WNT5B in the tumor microenvironment unlike its paralog WNT5A, which is known to be secreted by the stroma.^25,26^

In contrast, cyclin-dependent kinase regulatory subunit 1 (CKS1B) had the highest enrichment in cancer cell lines for all tissue types (Figure 2G). CKS1B is defined by Vesiclepedia as being detected in extracellular vesicles, where colorectal cancer SW620 was one of cell line models studied and is also on our panel.^27^ The functional role of exosome-derived CKS1B has not been defined, but CKS1B overexpression has been associated with resistance to proteasome inhibitor bortezomib,^28^ so CKS1B in extracellular vesicles could have a potential role in influencing drug response.

Functional enrichment of the CAF-enriched secreted proteins highlighted the known importance of CAFs in regulating the extracellular matrix^29^ (Figure 2H). Furthermore, there was enrichment of the regulation of vascular associated smooth muscle cells, immune response, and cell migration, which may illustrate the potential interplay between stromal cells and cancer cells that may be critically mediated by CAF-enriched secreted proteins.

### Identifying Potential Drug Resistance and Sensitivity in Cancer Cells Incubated with CAF CM

**Figure 3:**
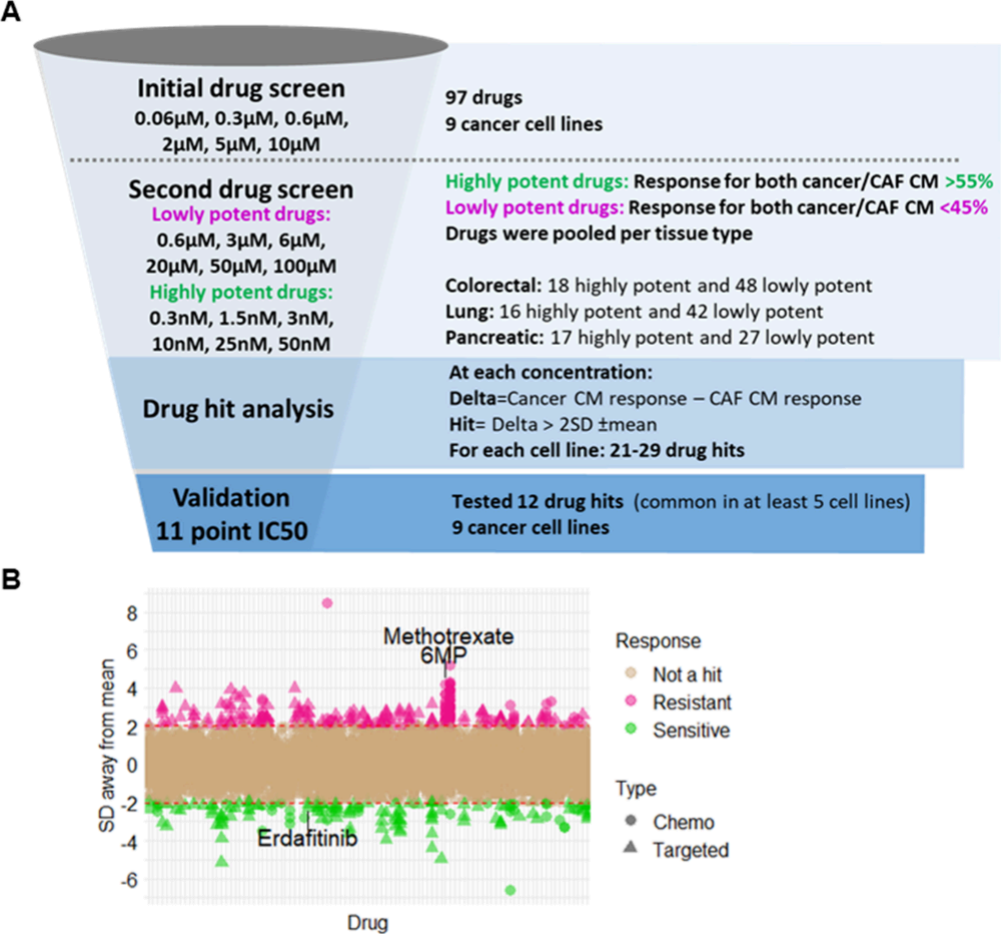
Assessing the impact of cancer-associated fibroblast (CAF) secreted proteins on drug response. (A) The drug screen comprised of two parts. For the initial screen, all nine cancer cell lines incubated in the appropriate conditioned media (CM) were treated with 97 different drugs at concentrations of 0.06, 0.3, 0.6, 2, 5, 10 μM. Response was calculated as % inhibition. In the second screen, drugs where the response was not in the optimal range in the initial screen were reassessed. Low potency drugs are where the response for both cancer and CAF CM was <45% while highly potent drugs are where the response for both cancer and CAF CM was >55%. Twelve drug hits were common in at least five cell lines (irrespective of directionality), and an 11 point IC_50_ curve was generated for these 12 drugs for all nine cancer cell lines in the drug validation stage. (B) Distribution of how many standard deviations (SD) are away from the mean for all the drugs and concentrations analyzed for the whole drug screen. Brown = not a hit, pink = CAF conditioned media (CM) mediated resistant drug hits, green = CAF CM mediated sensitive drug hits. Circle = chemotherapy (chemo) drug, triangle = targeted therapy drug.

To assess the impact of CAF secreted proteins on drug response, we investigated the response to 97 drugs (27 chemotherapy and 70 targeted therapy) in the nine cancer cell lines incubated with cancer or tissue matched CAF CM (Figure 3A). Differential drug hits mediated by CAF CM were identified if the difference between the cancer CM and CAF CM response (delta) was more than 2 standard deviations from the mean delta of all drugs at that specific concentration in a cancer cell line (Figure 3B).

With each cell line, 21–29 drugs with differential drug response in CAF CM compared to cancer CM were identified (Supplementary Table 4). In total, drug resistance mediated by CAF CM was more frequent than drug sensitivity with all the cell lines combined, where 114 cases had resistance with CAF CM, while 99 cases had sensitivity with CAF CM. There were 18 cases where the drug hit in the same cell line had both CAF CM mediated sensitivity and resistance defined at different concentrations. Together 82 hits were identified across the panel of *KRAS* mutant-cancer cells, which was comprised of 24 chemotherapy drugs (out of 27 = 88.8%) in at least one cell line and 58 targeted therapy drugs (out of 70 = 82.9%) in at least one cell line. There was not a universal differential drug response across all cell lines, which highlights the complexity in treating *KRAS* mutant-cancer where the same drug treatment can have different effects in different *KRAS* mutant-cancers.^30^

The differential drug response hits were ranked by the number of cell lines they had in common irrespective of the response directionality (sensitivity or resistance), and 12 drug hits, which were common in at least five cell lines, were further investigated. Antifolate methotrexate had the greatest consistent resistance with CAF CM in four out of the nine cancer cell lines (NSCLC H1792 and H23 and pancreatic cancer CAPAN1 and DANG; Supplementary Figure 2A), and purine analog 6-mercaptopurine (6MP) was also found to be consistently resistant with CAF CM in NSCLC H1792 and pancreatic cancer CAPAN1 (Supplementary Figure 2B). In contrast, CAF CM mediated sensitivity to fibroblast growth factor receptor (FGFR) inhibitor erdafitinib was validated in NSCLC H1792, but CAF CM mediated erdafitinib sensitivity in NSCLC H23 and CAF CM mediated erdafitinib resistance colorectal cancer H747 and SW620 were not consistently reproduced from the drug screen (Supplementary Figure 2C).

**Figure 4:**
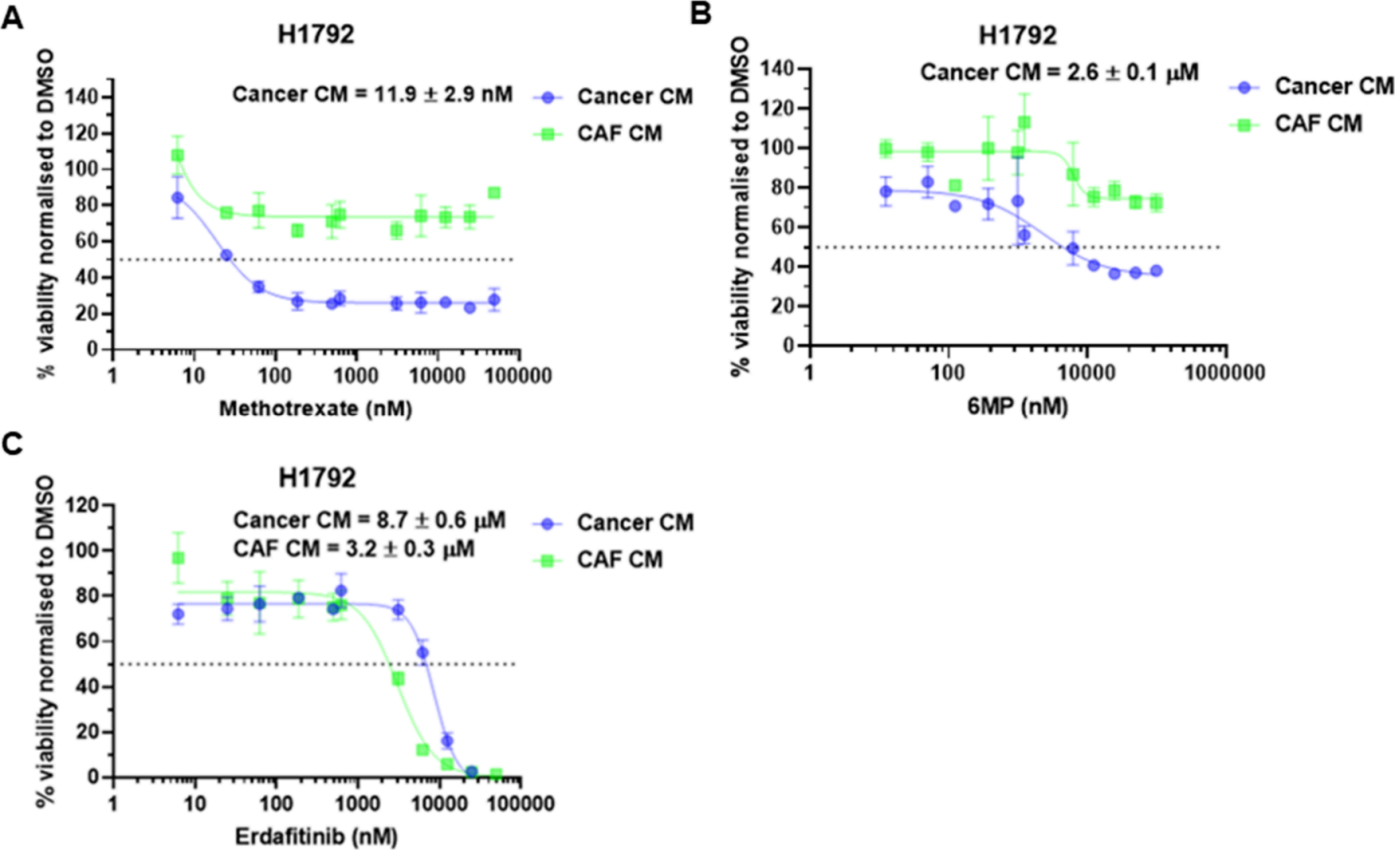
Significant differential drug responses with cancer-associated fibroblast (CAF) secreted proteins in lung cancer H1792 cell lines. (A) Methotrexate response curve, (B) 6-mercaptopurine (6MP) response curve, (C) erdafitinib response curve for lung cancer H1792. Response curves are plotted as the mean ± standard error of the mean (SEM) of three technical repeats and are representative of three independent experiments.

Interestingly, NSCLC H1792 was a cell line where there was consistent CAF CM mediated resistance to methotrexate (Figure 4A) and 6MP (Figure 4B) and erdafitinib sensitivity (Figure 4C). The Genomics of Drug Sensitivity in Cancer (GDSC) database illustrated that H1792 cells alone have a methotrexate IC_50_ of 9.5 nM, which is similar to our drug screen with cancer cells in cancer CM (11.9 nM).^31^ However, it was noted that erdafitinib and 6MP were not investigated in the GDSC screens.

## Discussion

The tumor microenvironment forms a significant portion of the tumor mass and is an abundant source of secreted proteins that can influence cancer cell signaling and so potentially drug response.^1,2^ Here, we present the direct comparative analysis of the CAF and cancer cell secretome where we identified the differentially expressed secreted proteins and studied the functional consequences of the CAF secretome by undertaking a bespoke drug screen of 97 anticancer drugs.

Comparative analysis confirmed CAFs as a major source of secreted proteins, with the majority of the differentially expressed secreted proteins attributed to this cell type. Several of the identified highly expressed proteins in the CAF secretome compared to the cancer secretome are known CAF markers, such as FSTL1 and COL1A1,^22−24^ but WNT5B was identified as CAF-enriched and is a relatively understudied WNT factor in CAF research. It is possible that CAF-derived WNT5B may have similar effects as WNT5A, which is known to be highly secreted by stromal cells,^25,26^ since the two proteins have high sequence homology (80%).^32^ For instance, WNT5A regulates ABC transporters,^33^ and so CAF-derived WNT5B may promote resistance by promoting drug efflux through increasing transporter protein expression. However, WNT5B may also have unique functions because emerging studies have proposed that WNT5B has a distinct expression profile and role in mouse development and cell differentiation compared to WNT5A.^34−37^ Validation and full exploration of WNT5B function in CAFs were outside the scope of this paper, but the secretome profiling undertaken here is an important resource for researchers to define CAF secreted proteins that are differentially expressed from cancer cells.

To characterize the function of the CAF secretome, a drug screen was run in the 9 *KRAS* mutant-cancer cells grown in cancer or tissue matched CAF CM where 97 chemotherapeutic and targeted therapy agents were assessed. No drugs had the same differential response with CAF CM compared to cancer CM across all 9 *KRAS* mutant-cancer cell lines, suggesting that there is no single overarching mechanism of response to a given drug caused by CAF and so emphasizes the complexity of treating *KRAS* mutant-cancer cells. In the screen, the greatest change in sensitivity in CAF CM conditions was that of drug resistance to methotrexate and 6MP, which was consistently observed in 4/9 cell lines and 2/9 cell lines on the panel, respectively. Methotrexate and 6MP resistance in certain cell lines may be due to specific advantageous cell signaling pathways that promote survival or compensate for altered metabolism in response to CAF-secreted proteins. For instance, as mentioned previously, CAF-derived WNT5B may increase ABC transporter expression and drug efflux in a similar manner to what has been reported for WNT5A.^33^ Future experiments will elucidate the mechanism of action behind CAF-mediated resistance in certain cell lines.

Interestingly, H1792 lung cancer cells had consistent CAF CM mediated resistance to methotrexate and 6MP and erdafitinib sensitivity. It is thought that *KRAS* mutant-lung cancer cells are sensitive to antifolate treatment^38^ so methotrexate resistance we observed with CAF CM in H1792 cells illustrates that certain observations may be missed with routine screening of cancer cells alone. Methotrexate resistance mediated by CAF CM has been investigated by Zhang et al. in CRC cells,^39^ where they identified caudal-related homeobox 2 (CDX2) and hephaestin (HEPH) downregulation in methotrexate resistant CRC cells and miR-24–3-p in the CAF exosomes promoted higher resistance compared to normal fibroblast exosomes.

Conversely, 6MP resistance and sensitivity to the FGFR inhibitor erdafitinib associated with CAF CM have not been investigated before. The exploration and validation into the exact mechanism of action behind these differential drug responses are outside the scope of this paper. Of the FGF ligands, only FGF19 and FGF2 were identified in the secretome analysis, and FGF19 levels between CAF CM and cancer CM were not significantly different. However, FGF2 levels were significantly 1.75-fold higher in CAF CM compared to cancer CM and so could be a potential driver of erdafitinib sensitivity in H1792 cells. It could also be possible that the enrichment of ECM proteins, including collagen and fibronectin, in the CAF CM compared to cancer CM could potentiate cancer cells to erdafitinib sensitivity given that ECM proteins has been reported to upregulate FGFR signaling.^40,41^ Future work on whether CAF-derived secreted ECM proteins influences FGFR signaling and erdafitinib sensitivity in cancer cells may help widen the use of FGFR inhibitors, which is currently used predominately for the treatment of cancer patients which harbor FGFR alterations.^42,43^

In conclusion, our study compares directly for the first time CAF-derived secreted proteins and the cancer-derived secreted proteins using mass spectrometry, and our 97 anticancer drug library revealed that the CAF secretome caused differing drug responses, which may be missed on routine drug screens involving cancer cells alone and so offers new insights on the use of existing anticancer drugs. Therefore, the characterization of the CAF and cancer secretome in association with cancer drug response is an important resource in highlighting the CAF-enriched secreted proteins and how they may affect drug response.

## Data Availability

The mass spectrometry secretome data have been deposited to the ProteomeXchange Consortium via the PRIDE^44^ partner repository with the data set identifier: PXD048307.

## Supporting Information Available

(Figure S1) The viability of cells used for secretome analysis; (Figure S2) validation of drug screen hits; (Table S1) list of anticancer drugs in the drug screen panel; (Table S2) list of identified secreted or transmembrane proteins; (Table S3) significant differentially expressed secreted proteins between cancer-associated fibroblasts and cancer cell lines; (Table S4) list of drug hits (PDF)
