# Supplementary material for: Secretome of Cancer-Associated Fibroblasts (CAFs) Influences Drug Sensitivity in Cancer Cells
Source: J Proteome Res. 2024 May 20;23(6):2160–8. doi: 10.1021/acs.jproteome.4c00112 (PMC11165579; doi:10.1021/acs.jproteome.4c00112)
Supplement: Supplementary file 1 — pr4c00112_si_001.pdf [file pr4c00112_si_001.pdf]

Secretome of cancer-associated fibroblasts (CAFs) influences drug sensitivity in cancer cells

Rachel Lau[1], Lu Yu[2], Theodoros I. Roumeliotis[2], Adam Stewart[1], Lisa Pickard[1], *Jyoti S. Choudhary[2] and *Udai Banerji[1]

1. Clinical Pharmacology and Adaptive Therapy Group, The Institute of Cancer Research and The Royal Marsden NHS Foundation Trust, London, SM2 5PT, UK
2. Functional Proteomics Group, Chester Beatty Laboratories, The Institute of Cancer Research, London, SW3 6JB, UK

*Corresponding Authors

Contents

Fig S1 The viability of cells used for secretome analysis 3

Fig S2 Validation of drug screen hits.. 4

Table S1 List of anti-cancer drugs in the drug screen panel 5

Table S2 List of identified secreted or transmembrane proteins 7

Table S3 Significant differentially expressed secreted proteins between cancer-associated fibroblasts and cancer cell lines 125

Table S4 List of drug hits 131

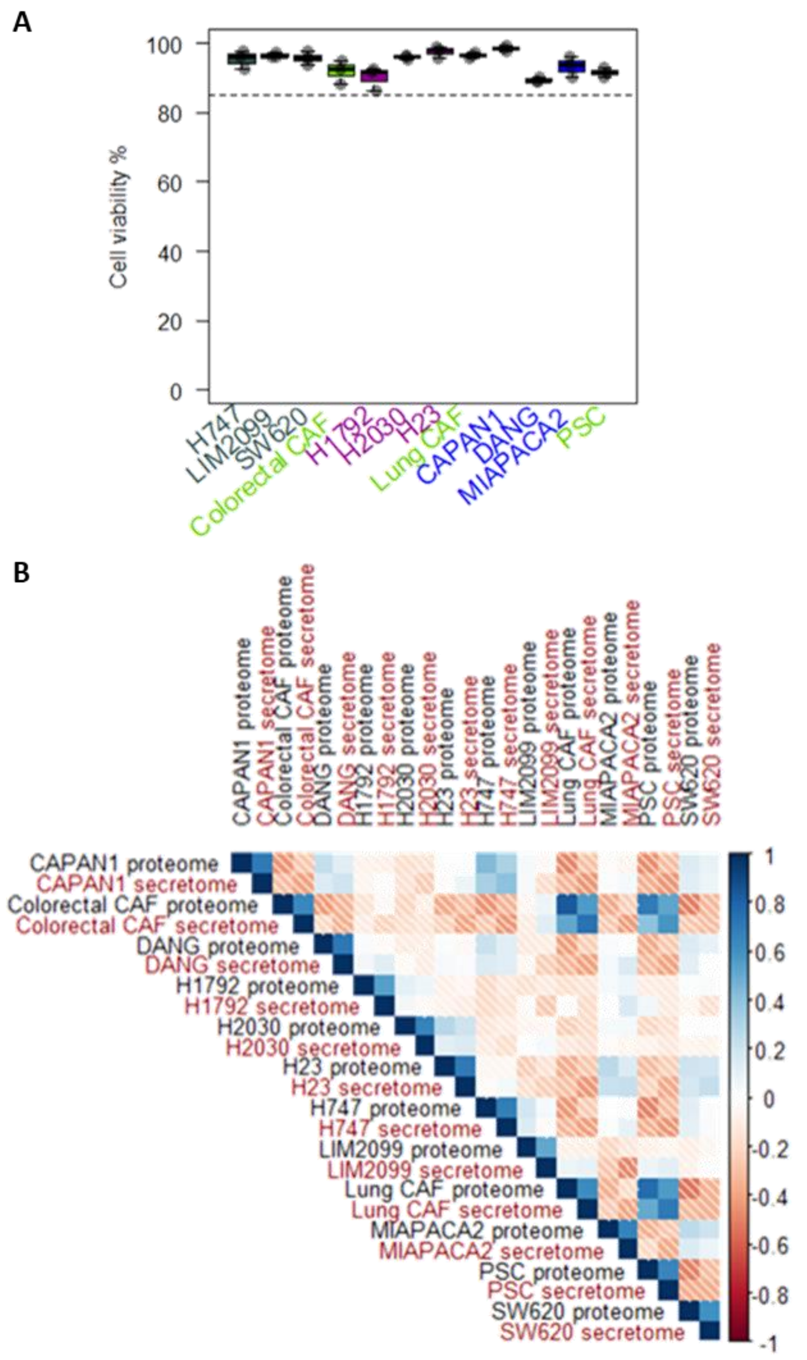

Fig S1 The viability of cells used for secretome analysis

(A) Boxplot of the cell viability measured by Trypan Blue upon conditioned media harvesting after 24 hour serum deprivation. All cell lines exhibited cell viability above 85%. **(B)** Correleogram between the abundance of the secreted or transmembrane proteins identified in our secretome analysis and its corresponding expression in our proteome analysis of cells cultured under normal 10% serum conditions.

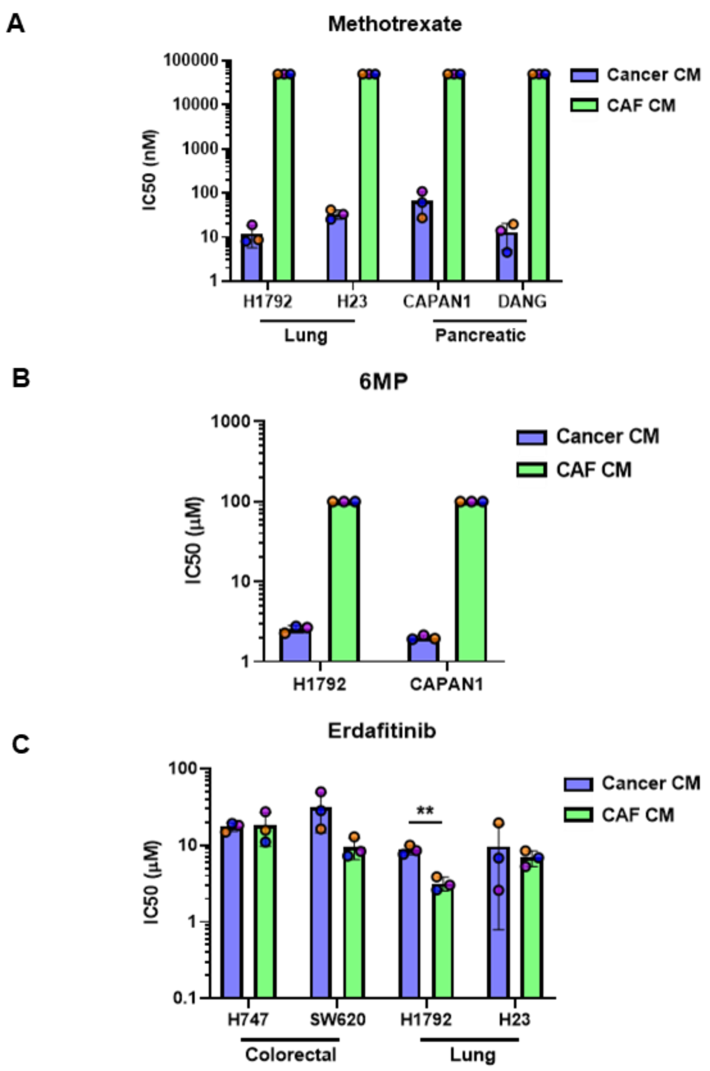

Fig S2 Validation of drug screen hits.

(A) Barplot of the methotrexate IC50s in lung cancer H1792 and H23 and in pancreatic cancer CAPAN1 and DANG with cancer or CAF CM. IC50s could not be determined in cells with CAF CM and thus are plotted at the maximum concentration investigated (50,000 nM). **(B)** Barplot of the 6MP IC50s in lung cancer H1792 and in pancreatic cancer CAPAN1 with cancer or CAF CM. IC50s could not be determined in cells with cancer CM and thus are plotted at the maximum concentration investigated (100 µM). (C) Barplot of the erdafitinib IC50s in colorectal cancer H747 and SW620 and in lung cancer H1792 and H23 with cancer or CAF CM.

Table S1 List of anti-cancer drugs in the drug screen panel

Class	Drugs
AKT inhibitor	Capivasertib (AZD5363), Ipatasertib (GDC-0068, RG7440)
ALK inhibitor	Certinib (LDK378), alectinib (CH5424802), Crizotinib hydrochloride
Alkylating agent	Ifosfamide, Dacarbazine, Oxaliplatin, Temozolomide,
Androgen receptor inhibitor	Enzalutamide (MDV3100), Flutamide
Anti-folate	Methotrexate, Pemetrexed
Anti-inflammatory	Prednisolone
Antimicrotubule	Vincristine sulfate, Paclitaxel (Taxol), Docetaxel, Vinorelbine
Ataxia telangiectasia and RAD3 related (ATR) inhibitor	Ceralasertib (AZD6738), Elimusertib (BAY1895344)
Tyrosine-protein kinase receptor UFO (AXL) inhibitor	Bemcentinib (R428)
B-cell lymphoma 2 (BCL2) inhibitor	Venetoclax (ABT-199)
Bromodomain and extra-terminal domain (BET) inhibitor	Molibresib (I-BET-762)
BRAF inhibitor	Encorafenib, Dabrafenib (GSK2118436)
Bruton Tyrosine Kinase (BTK) inhibitor	Ibrutinib (PCI-32765)
CDK4/6 inhibitor	Ribociclib (LEE011), Palbociclib (PD0332991)
Checkpoint kinase 1 (CHK1) inhibitor	CCT245737 (SRA737), Prexasertib (LY2606368 HCl)
DNA methylation inhibitor	Decitabine (NSC127716, 5AZA-CdR), 5-Azacytidine
EGFR inhibitor	Gefitinib (ZD1839), Osimertinib
Endothelin receptor inhibitor	Zibotentan (ZD4054)
ERK inhibitor	SCH772984
Focal adhesion kinase (FAK) inhibitor	Defactinib
Fibroblast growth factor receptor (FGFR) inhibitor	AZD4547, Erdafitinib
Fms-like tyrosine kinase 3 (FLT3) inhibitor	Midostaurin (PKC412)
Human homolog of mouse double minute 2 (HDM2) inhibitor	HDM201
Human epidermal growth factor receptor 2 (HER2) inhibitor	Lapatinib, Neratinib (HKI-272)
Histone deacetylase (HDAC) inhibitor	Vorinostat (SAHA, MK0683), Panobinostat (LBH589)
Heat shock protein 90 (HSP90) inhibitor	17-AAG (KOS953), AUY922 (NVP-AUY922)
Isocitrate dehydrogenase 1 (IDH1) inhibitor	Ivosidenib (AG-120)
Isocitrate dehydrogenase 2 (IDH2) inhibitor	Enasidenib (AG-221)

Insulin growth factor receptor (IGFR) inhibitor	Linsitinib
Immunomodulatory	Lenalidomide (CC-5013), Thalidomide
Inhibitor of apoptosis proteins (IAPs)	LCL161
JAK inhibitor	Ruxolitinib (INCB018424), Fedratinib (TG101348, SAR302503)
$KRAS^{G12C}$ inhibitor	Sotorasib (AMG-510)
MEK inhibitor	Trametinib (GSK1120212), AZD6244 (Selumetinib)
Monopolar spindle (MPS) inhibitor	Empesertib (BAY1161909)
Mammalian target of Rapamycin (MTOR) inhibitor	Everolimus (RAD001), vistusertib (AZD2014)
Multi-target RTK inhibitor	Dasatinib (BMS-354825), Regorafenib, Nintedanib (BIBF 1120), Imatinib (STI571)
Poly (ADP-ribose) polymerase (PARP) inhibitor	Veliparib (ABT-888), Niraparib (MK-4827), Olaparib (AZD2281, Ku-0059436)
Platelet derived growth factor receptor (PDGFR) inhibitor	Crenolanib (CP-868596)
PI3K inhibitor	Pictilisib (GDC-0941), Alpelsib (BYL-719), GSK2636771
Proteosome inhibitor	Bortezomib (PS-341)
Purine analog	Mercaptopurine (6MP), Fludarabine
Pyrimidine analog	Fluorouracil (Adrucil), Gemcitabine
RAF/MEK inhibitor	RO5126766 (CH5126766)
Retinoid X receptor agonists	Bexarotene
Rho-associated protein kinase 1 (ROCK) inhibitor	Fasudil (HA-1077) HCl
Selective oestrogen receptor modulator (SERM)	Tamoxifen
Smoothened inhibitor	Vismodegib (GDC-0449), Erismodegib (NVP-DE225, LDE225)
Stimulator of interferon genes (STING) agonist	Vadimezan (DMXAA)
TGF-β receptor (TGFBR) inhibitor	Galunisertib (LY2157299)
Topoisomerase I inhibitor	Topotecan, SN-38 (7-Ethyl-10-hydroxycamptothecin)
Topoisomerase II inhibitor	Etoposide, Epirubicin HCl, Doxorubicin
Tropomyosin receptor kinase (TRK) inhibitor	Entrectinib, Larotrectinib (LOXO-101)
Vascular endothelial growth factor (VEGFR) inhibitor	Sunitinib, Cediranib (AZD217), Lenvatinib (E7080)
WEE1 inhibitor	Adavosertib (MK-1775)
WNT/β-catenin inhibitor	IWR-1-endo

Table S2 List of identified secreted or transmembrane proteins

Proteins are annotated by different software and databases: SignalP, secretomeP, TMHMM, Cell Surface Protein Atlas (CSPA), surfaceome, FANTOM5 and vesiclepedia

Accession	Gene.name	SignalP	SecretomeP	TMHMM	CSPA	Surfaceome	FANTOM5	Vesiclepedia
ENST00000359671_NCI-H23_Mis:G1188V	FN1	Yes			Yes		Yes	
P02751	FN1	Yes			Yes		Yes	
P06733	ENO1				Yes			
P08123	COL1A2	Yes			Yes		Yes	Yes
P60709	ACTB				Yes			Yes
P63261	ACTG1							Yes
P02452	COL1A1	Yes			Yes		Yes	Yes
P08670	VIM				Yes		Yes	
P04406	GAPDH				Yes			
P12111	COL6A3	Yes			Yes		Yes	Yes
P11142	HSPA8				Yes			
P08238	HSP90AB1				Yes			
P21333	FLNA				Yes			
Q99715	COL12A1	Yes			Yes			Yes
ENST00000472056_NCI-H23_Mis:T117M	COL6A3	Yes			Yes		Yes	Yes

P35579	MYH9				Yes			
O43707	ACTN4				Yes			Yes
P07996	THBS1	Yes			Yes		Yes	
P98160	HSPG2	Yes			Yes		Yes	
P04075	ALDOA							Yes
P07900	HSP90AA1				Yes		Yes	
P14618	PKM				Yes		Yes	
P04264	KRT1				Yes			
Q00610	CLTC				Yes			Yes
P02545	LMNA				Yes			
P68032	ACTC1							Yes
P0DP25			Yes					Yes
O00468	AGRN	Yes			Yes		Yes	
P60174	TPI1				Yes			
P68133	ACTA1							Yes
P68104	EEF1A1				Yes			
P12814	ACTN1				Yes			Yes
P11021	HSPA5	Yes						
P11047	LAMC1	Yes			Yes		Yes	
Q15582	TGFBI	Yes						
P07942	LAMB1	Yes			Yes		Yes	
ENST00000316292_NCI-H23_Mis:R239S	EEF1A1				Yes			

P09211	GSTP1						Yes	
P06744	GPI				Yes		Yes	
P02461	COL3A1	Yes			Yes		Yes	Yes
P07437	TUBB				Yes			
P00750	PLAT	Yes			Yes		Yes	
P10809	HSPD1				Yes			
P68371	TUBB4B				Yes			
P35555	FBN1	Yes			Yes		Yes	
P08253	MMP2	Yes					Yes	
P06748	NPM1		Yes					
P12109	COL6A1	Yes			Yes		Yes	Yes
P05783	KRT18		Yes					
P05067	APP	Yes		Yes		Yes	Yes	Yes
P05121	SERPINE1	Yes					Yes	
P06396	GSN	Yes						
P24821	TNC	Yes			Yes		Yes	
P04350	TUBB4A				Yes			
Q16363	LAMA4	Yes			Yes		Yes	
P07237	P4HB	Yes					Yes	
P68366	TUBA4A				Yes			
O15230	LAMA5	Yes			Yes		Yes	
P19022	CDH2	Yes		Yes	Yes	Yes		Yes

Q86UP2	KTN1			Yes				
P80188	LCN2	Yes					Yes	
P22314	UBA1				Yes			
P14625	HSP90B1	Yes			Yes			
P13667	PDIA4	Yes						
O94985	CLSTN1	Yes		Yes		Yes		
P62979	RPS27A		Yes					
P30101	PDIA3	Yes						
P03956	MMP1	Yes					Yes	
Q15063	POSTN	Yes			Yes			
Q08380	LGALS3BP	Yes			Yes		Yes	
P07737	PFN1				Yes			
Q6YHK3	CD109	Yes			Yes	Yes		
Q02818	NUCB1	Yes						
P23528	CFL1		Yes					Yes
ENST00000508830_LIM2099_Mis:D532N	CAST							Yes
Q92626	PXDN	Yes		Yes	Yes			
O00391	QSOX1	Yes		Yes	Yes	Yes		
P07585	DCN	Yes			Yes		Yes	Yes
P27797	CALR	Yes			Yes		Yes	Yes
ENST00000338252_LIM2099_Mis:D436N	CAST							Yes
P20810	CAST							Yes

P40926	MDH2		Yes					
P07602	PSAP	Yes			Yes		Yes	
P53396	ACLY							Yes
P12956	XRCC6				Yes			
P05997	COL5A2	Yes			Yes		Yes	Yes
Q16531	DDB1							Yes
Q00839	HNRNPU				Yes			
P04083	ANXA1						Yes	Yes
ENST00000444376_CAPAN-1_Mis:P658S	HNRNPU				Yes			
P10909	CLU	Yes			Yes			Yes
P13521	SCG2	Yes		Yes				
P27348	YWHAQ				Yes			
P61978	HNRNPK				Yes			
P09486	SPARC	Yes						
P02786	TFRC		Yes	Yes	Yes			
Q06481	APLP2	Yes		Yes	Yes	Yes		Yes
P50991	CCT4				Yes			
P07355	ANXA2		Yes					Yes
P31939	ATIC							Yes
Q14517	FAT1	Yes		Yes	Yes	Yes		
P08581	MET	Yes		Yes	Yes	Yes		

P37802	TAGLN2		Yes		Yes			
P09493	TPM1		Yes					
P01011	SERPINA3	Yes			Yes			Yes
P09874	PARP1							Yes
P12110	COL6A2	Yes			Yes		Yes	Yes
P09972	ALDOC							Yes
P07339	CTSD	Yes			Yes			Yes
O00410	IPO5		Yes					
Q15393	SF3B3		Yes					
P60842	EIF4A1		Yes					
P23284	PPIB	Yes		Yes	Yes			
Q9P2E9	RRBP1			Yes				
P50454	SERPINH1	Yes			Yes			Yes
P02671	FGA	Yes			Yes		Yes	
P27708	CAD							Yes
Q12805	EFEMP1	Yes			Yes		Yes	
P00352	ALDH1A1							Yes
P14543	NID1	Yes					Yes	
P02675	FGB	Yes			Yes		Yes	
P15121	AKR1B1							Yes
P49588	AARS				Yes			Yes
P26599	PTBP1				Yes			

Q02809	PLOD1	Yes			Yes			
Q9Y4K0	LOXL2	Yes						
Q9Y4L1	HYOU1	Yes		Yes	Yes			
P40227	CCT6A							Yes
Q9BUF5	TUBB6				Yes			
P04275	VWF	Yes			Yes		Yes	
P16035	TIMP2	Yes					Yes	
O00299	CLIC1							Yes
P04792	HSPB1		Yes					
P43490	NAMPT						Yes	
P10586	PTPRF	Yes		Yes	Yes	Yes		
O43852	CALU	Yes			Yes			Yes
Q8NBJ4	GOLM1			Yes	Yes			
P08572	COL4A2	Yes		Yes			Yes	Yes
P07093	SERPINE2	Yes			Yes		Yes	
O14980	XPO1		Yes					
P31949	S100A11		Yes					
ENST00000397763_CAPAN-1_Mis:L693F	COL6A2	Yes			Yes		Yes	Yes
Q92520	FAM3C			Yes				
P35556	FBN2	Yes		Yes	Yes			
P05387	RPLP2	Yes			Yes			

P23526	AHCY							Yes
P49915	GMPS				Yes			
Q12841	FSTL1	Yes			Yes			
P21810	BGN	Yes			Yes		Yes	Yes
P16949	STMN1		Yes					
P55060	CSE1L							Yes
P21980	TGM2						Yes	
Q14766	LTBP1	Yes			Yes		Yes	
P05204	HMGN2		Yes					
Q14112	NID2	Yes			Yes		Yes	
P63010	AP2B1		Yes					Yes
Q14118	DAG1	Yes		Yes	Yes	Yes		Yes
Q15293	RCN1	Yes			Yes			
P14174	MIF		Yes					
P62701	RPS4X		Yes					
P08603	CFH	Yes			Yes		Yes	
P55058	PLTP	Yes			Yes		Yes	
Q14563	SEMA3A	Yes			Yes		Yes	
P35221	CTNNA1							Yes
P02679	FGG	Yes		Yes			Yes	
Q9UQ80	PA2G4				Yes			
P51884	LUM	Yes			Yes			

Q02388	COL7A1	Yes			Yes		Yes	Yes
Q6UVK1	CSPG4	Yes		Yes	Yes	Yes		Yes
P10451	SPP1	Yes					Yes	
Q14697	GANAB	Yes		Yes				
P35052	GPC1	Yes			Yes	Yes		
O60568	PLOD3	Yes			Yes			
Q92563	SPOCK2	Yes						
P36955	SERPINF1	Yes			Yes			
P47895	ALDH1A3							Yes
P11717	IGF2R	Yes		Yes	Yes	Yes		
Q9BRK5	SDF4	Yes		Yes				
Q13740	ALCAM	Yes		Yes	Yes	Yes		Yes
P39023	RPL3				Yes			
Q14847	LASP1		Yes					
Q05682	CALD1							Yes
Q16706	MAN2A1			Yes	Yes			
P01024	C3	Yes			Yes		Yes	Yes
P09429	HMGB1						Yes	
Q92820	GGH	Yes						
P61247	RPS3A		Yes					
P08865	RPSA		Yes					
P00736	C1R	Yes						

ENST00000237623_NCI-H23_Mis:R153M	SPP1	Yes					Yes	
ENST00000592741_NCI-H747_Mis:E209K	PRKCSH	Yes						
P01033	TIMP1	Yes			Yes		Yes	
O14672	ADAM10	Yes		Yes	Yes	Yes	Yes	Yes
P17661	DES		Yes					
P10124	SRGN	Yes						
Q9UGM3	DMBT1	Yes						
P14868	DARS							Yes
P13798	APEH							Yes
P80303	NUCB2	Yes		Yes				
P16070	CD44	Yes		Yes	Yes	Yes		Yes
P14314	PRKCSH	Yes			Yes			
P15559	NQO1		Yes					
P17844	DDX5							Yes
Q9UHD8	SEPTIN9		Yes					
Q14767	LTBP2	Yes						
O15240	VGF	Yes						
P35998	PSMC2		Yes					
P53634	CTSC	Yes			Yes			Yes
P05120	SERPINB2				Yes			
P30044	PRDX5		Yes					

O00469	PLOD2	Yes			Yes			
Q16270	IGFBP7	Yes						
P00441	SOD1		Yes					
Q15113	PCOLCE	Yes						
P29692	EEF1D				Yes			
Q14019	COTL1		Yes					
P24592	IGFBP6	Yes						
Q13753	LAMC2	Yes			Yes		Yes	
P00749	PLAU	Yes			Yes		Yes	
Q92896	GLG1	Yes		Yes	Yes			
P19021	PAM	Yes		Yes		Yes		
P15880	RPS2		Yes					
Q9UBP4	DKK3	Yes			Yes			
P05556	ITGB1	Yes		Yes	Yes	Yes		
P48444	ARCN1							Yes
Q16610	ECM1	Yes					Yes	
O00622	CYR61	Yes					Yes	
Q14126	DSG2	Yes		Yes	Yes	Yes		
P35442	THBS2	Yes			Yes		Yes	
Q9NZ08	ERAP1	Yes			Yes			
P20908	COL5A1	Yes			Yes		Yes	Yes
Q16787	LAMA3	Yes			Yes		Yes	

P17655	CAPN2							Yes
P28074	PSMB5		Yes					
O75821	EIF3G		Yes					
Q9Y520	PRRC2C				Yes			
P27695	APEX1		Yes					Yes
P24043	LAMA2	Yes			Yes		Yes	
P16152	CBR1		Yes					Yes
P30086	PEBP1		Yes					
P23142	FBLN1	Yes					Yes	
P09871	C1S	Yes						Yes
Q9Y281	CFL2		Yes					Yes
Q9HC38	GLOD4		Yes					
P53618	COPB1							Yes
Q15084	PDIA6	Yes						
Q16181	SEPTIN7							Yes
Q10567	AP1B1							Yes
O75368	SH3BGRL		Yes					
P52907	CAPZA1							Yes
P13497	BMP1	Yes			Yes			Yes
P07686	HEXB			Yes	Yes			
P67809	YBX1		Yes		Yes			
Q13185	CBX3		Yes					

O14786	NRP1	Yes		Yes	Yes	Yes		
P26022	PTX3	Yes						
Q04828	AKR1C1							Yes
O75326	SEMA7A	Yes			Yes	Yes	Yes	
O43405	COCH	Yes						
O95782	AP2A1							Yes
Q86X29	LSR			Yes				
P12830	CDH1	Yes		Yes	Yes	Yes	Yes	Yes
ENST00000602122_NCI-H1792_Mis:D451N	LSR			Yes				
P40121	CAPG							Yes
Q16643	DBN1							Yes
Q9NZV1	CRIM1	Yes		Yes	Yes	Yes		
Q92499	DDX1							Yes
Q9NYU2	UGGT1	Yes		Yes	Yes			
P06865	HEXA	Yes			Yes			
P01034	CST3	Yes						Yes
Q9BTY2	FUCA2	Yes						
Q13442	PDAP1						Yes	
P21399	ACO1							Yes
Q86UE4	MTDH			Yes				
O60911	CTSV	Yes			Yes			Yes

Q8NBP7	PCSK9	Yes					Yes	
P07108	DBI							Yes
Q92823	NRCAM	Yes		Yes	Yes	Yes		
P17931	LGALS3		Yes					
P22692	IGFBP4	Yes					Yes	
P07858	CTSB	Yes						Yes
P62249	RPS16		Yes					
P62333	PSMC6		Yes					
P42167	TMPO			Yes				
P00492	HPRT1		Yes					
Q15436	SEC23A		Yes					
P62328	TMSB4X		Yes					
P42330	AKR1C3		Yes					
P31431	SDC4	Yes		Yes				
P30048	PRDX3		Yes					
P30040	ERP29	Yes						
P06576	ATP5B							Yes
Q9UI42	CPA4	Yes						
P62906	RPL10A		Yes					
P13611	VCAN	Yes			Yes		Yes	Yes
Q08629	SPOCK1	Yes						

ENST00000361796_NCI-H23_Mis:G830R	CTNND1							Yes
Q9HB71	CACYBP		Yes					
P08195	SLC3A2		Yes	Yes	Yes	Yes		
P10321	HLA-C	Yes		Yes	Yes	Yes	Yes	
P15144	ANPEP			Yes	Yes	Yes		Yes
P08758	ANXA5							Yes
P54819	AK2		Yes					Yes
P27635	RPL10		Yes					
Q14203	DCTN1							Yes
Q13443	ADAM9	Yes		Yes	Yes	Yes	Yes	
Q13162	PRDX4	Yes						
O75787	ATP6AP2	Yes		Yes				
P00533	EGFR	Yes		Yes	Yes	Yes		
Q99985	SEMA3C	Yes			Yes		Yes	
Q9BWD1	ACAT2		Yes					Yes
P39060	COL18A1	Yes					Yes	
P16989	YBX3		Yes					
P46778	RPL21		Yes		Yes			
P10646	TFPI	Yes			Yes	Yes	Yes	
P07384	CAPN1							Yes
O94907	DKK1	Yes					Yes	

Q08043	ACTN3							Yes
P30520	ADSS							Yes
P22676	CALB2							Yes
O00154	ACOT7		Yes					
P09012	SNRPA		Yes					
P38919	EIF4A3		Yes					
Q08257	CRYZ							Yes
O76021	RSL1D1				Yes			
O95336	PGLS		Yes					
O14737	PDCD5		Yes					
Q9Y5Y6	ST14		Yes	Yes	Yes			
P52565	ARHGDIA							Yes
O00584	RNASET2	Yes						
P49589	CARS							Yes
Q76M96	CCDC80	Yes			Yes			
Q92876	KLK6	Yes						
P05023	ATP1A1			Yes	Yes	Yes		Yes
P00966	ASS1							Yes
P01009	SERPINA1	Yes			Yes		Yes	
Q8WVQ1	CANT1		Yes		Yes			
Q08174	PCDH1	Yes		Yes	Yes	Yes		
P33151	CDH5	Yes		Yes	Yes	Yes		Yes

P29279	CTGF	Yes					Yes	Yes
P48307	TFPI2	Yes			Yes			
P21281	ATP6V1B2							Yes
O00754	MAN2B1	Yes			Yes			
O43278	SPINT1	Yes		Yes			Yes	
P83916	CBX1		Yes					
P08708	RPS17		Yes					
P62081	RPS7		Yes					
P47756	CAPZB							Yes
P62917	RPL8		Yes					
P26447	S100A4		Yes					
Q12931	TRAP1		Yes		Yes			
P29966	MARCKS				Yes			
P04439	HLA-A	Yes		Yes	Yes	Yes	Yes	
P09668	CTSH	Yes						Yes
Q15828	CST6	Yes						Yes
P16144	ITGB4	Yes			Yes	Yes		
P62316	SNRPD2		Yes		Yes			
P08254	MMP3	Yes						
P84103	SRSF3				Yes			
P01008	SERPINC1	Yes					Yes	Yes
P61916	NPC2	Yes						

Q8NBS9	TXNDC5	Yes						
P11766	ADH5							Yes
O00571	DDX3X							Yes
O43399	TPD52L2		Yes					
Q10471	GALNT2			Yes				
P53999	SUB1		Yes					
Q9NZM1	MYOF			Yes	Yes	Yes		
P47755	CAPZA2							Yes
P17301	ITGA2	Yes		Yes	Yes	Yes		
P49419	ALDH7A1							Yes
P62280	RPS11		Yes					
Q8NE71	ABCF1							Yes
Q96AY3	FKBP10	Yes			Yes			
ENST00000368207_SW620_Mis:V774I	PTPRK	Yes		Yes				
O43240	KLK10	Yes						
P08133	ANXA6							Yes
Q9NQR4	NIT2		Yes					
P50502	ST13		Yes					
O94973	AP2A2							Yes
P63208	SKP1		Yes					
O43854	EDIL3	Yes					Yes	
P21589	NT5E	Yes		Yes	Yes	Yes		

O96019	ACTL6A							Yes
Q15056	EIF4H		Yes					
P07711	CTSL	Yes			Yes			Yes
Q13332	PTPRS	Yes		Yes	Yes	Yes		
P54577	YARS						Yes	
Q9UJU6	DBNL		Yes					
Q99538	LGMN	Yes			Yes			
O00499	BIN1							Yes
Q9H299	SH3BGRL3		Yes					
P24593	IGFBP5	Yes						
P52566	ARHGDIB							Yes
P04040	CAT							Yes
P28799	GRN	Yes			Yes			
Q9H3G5	CPVL	Yes			Yes			
O15372	EIF3H		Yes					
Q9GZM7	TINAGL1	Yes						
Q13011	ECH1		Yes					
P01130	LDLR	Yes		Yes	Yes	Yes		
Q99674	CGREF1	Yes						
P61769	B2M	Yes					Yes	Yes
O95084	PRSS23	Yes			Yes		Yes	
P55268	LAMB2	Yes			Yes		Yes	

P50552	VASP		Yes				Yes	
P46779	RPL28				Yes			
P18754	RCC1		Yes					Yes
Q6NZI2	PTRF		Yes					
Q9UHI8	ADAMTS1	Yes						
P62140	PPP1CB		Yes					
P04062	GBA	Yes			Yes			
P09237	MMP7	Yes					Yes	
P43487	RANBP1		Yes					
P46108	CRK							Yes
P61956	SUMO2		Yes					
P26196	DDX6							Yes
Q14257	RCN2	Yes						
Q9P287	BCCIP		Yes					
Q9Y240	CLEC11A	Yes					Yes	
O43747	AP1G1							Yes
P30050	RPL12		Yes					
O43493	TGOLN2	Yes		Yes		Yes		
Q9NS15	LTBP3	Yes		Yes	Yes		Yes	
Q93063	EXT2		Yes	Yes				
P00813	ADA							Yes
P17936	IGFBP3	Yes			Yes			

O00193	SMAP		Yes					
Q4LDE5	SVEP1	Yes						
Q5JPE7	NOMO2	Yes						
O60462	NRP2	Yes		Yes	Yes	Yes		
Q14789	GOLGB1			Yes				
P51114	FXR1		Yes					
P55263	ADK							Yes
P15586	GNS	Yes		Yes	Yes			
P08243	ASNS							Yes
ENST00000306100_NCI-H2030_Mis:H619Q	FSTL5	Yes						
Q92743	HTRA1	Yes						
Q8NES3	LFNG	Yes		Yes				
P19320	VCAM1	Yes		Yes	Yes	Yes	Yes	
P18621	RPL17				Yes			
O75882	ATRN			Yes	Yes	Yes		
P02462	COL4A1	Yes					Yes	Yes
P16422	EPCAM	Yes		Yes	Yes	Yes		
P35222	CTNNB1		Yes					Yes
Q96HE7	ERO1L	Yes			Yes			
P06454	PTMA						Yes	
Q13421	MSLN	Yes		Yes	Yes	Yes		

Q09028	RBBP4		Yes					
Q15155	NOMO1	Yes						
P46783	RPS10		Yes					
P49746	THBS3	Yes			Yes			
P30085	CMPK1		Yes					
P62308	SNRPG		Yes					
P46109	CRKL		Yes					Yes
Q9NR45	NANS		Yes					
Q15818	NPTX1	Yes			Yes			
Q15366	PCBP2				Yes			
P78539	SRPX	Yes						
Q96AG4	LRRC59		Yes	Yes				
P0C0L4	C4A	Yes			Yes		Yes	Yes
O15031	PLXNB2	Yes			Yes	Yes		
Q9UBR2	CTSZ	Yes						Yes
P20290	BTF3							Yes
O43684	BUB3		Yes					
P62266	RPS23				Yes			
P30405	PPIF		Yes					
Q07954	LRP1	Yes		Yes	Yes	Yes		
P25398	RPS12		Yes					
P35659	DEK		Yes					

O00487	PSMD14		Yes					
Q02543	RPL18A		Yes					
Q13751	LAMB3	Yes			Yes		Yes	
P20962	PTMS		Yes					
P43251	BTD				Yes			Yes
P33316	DUT		Yes					
Q9NQC3	RTN4			Yes	Yes		Yes	
Q13404	UBE2V1		Yes					
P63313	TMSB10		Yes					
P01137	TGFB1	Yes			Yes		Yes	
P29317	EPHA2	Yes		Yes	Yes	Yes		
Q16576	RBBP7		Yes					
P60981	DSTN		Yes					
P06280	GLA	Yes			Yes			
Q96D15	RCN3	Yes			Yes			
P61204	ARF3							Yes
P38571	LIPA	Yes			Yes			
P22090	RPS4Y1		Yes					
Q8NCW5	APOA1BP	Yes						
P18065	IGFBP2	Yes						
P61088	UBE2N		Yes					
Q7Z7M9	GALNT5			Yes	Yes			

P61353	RPL27		Yes					
O75937	DNAJC8		Yes					
P23229	ITGA6	Yes		Yes	Yes	Yes		
P30046	DDT							Yes
Q96CW1	AP2M1							Yes
P02649	APOE	Yes					Yes	Yes
Q9Y696	CLIC4		Yes					
Q09328	MGAT5			Yes	Yes			
O76061	STC2	Yes						
P49189	ALDH9A1							Yes
P32004	L1CAM	Yes		Yes	Yes	Yes	Yes	
Q9Y5Z4	HEBP2		Yes					
Q9H910	HN1L		Yes					
P39019	RPS19		Yes				Yes	
P00751	CFB	Yes						Yes
Q15819	UBE2V2		Yes					
O00567	NOP56				Yes			
P25311	AZGP1	Yes					Yes	Yes
P45877	PPIC	Yes		Yes				
O00339	MATN2	Yes						
Q15904	ATP6AP1	Yes		Yes	Yes			Yes
Q03001	DST							Yes

Q96RW7	HMCN1	Yes			Yes			
Q13308	PTK7	Yes		Yes	Yes	Yes		
P22223	CDH3	Yes		Yes	Yes	Yes		Yes
P62829	RPL23		Yes					
P46937	YAP1		Yes					
Q9UKM9	RALY				Yes			
Q8NFW8	CMAS		Yes					
O60888	CUTA			Yes				
P63220	RPS21		Yes					
P30530	AXL	Yes		Yes	Yes	Yes		Yes
P10619	CTSA	Yes			Yes			
P08476	INHBA	Yes					Yes	
A0A1B0GUS4			Yes					
Q9UKZ9	PCOLCE2	Yes						
P19883	FST	Yes					Yes	
Q04837	SSBP1		Yes					
Q9H0B8	CRISPLD2	Yes		Yes				
O15212	PFDN6		Yes					
P13861	PRKAR2A		Yes					
P48723	HSPA13	Yes		Yes	Yes			
Q07020	RPL18				Yes			
P10253	GAA			Yes	Yes			

Q03154	ACY1							Yes
P04424	ASL							Yes
Q8IUX7	AEBP1	Yes			Yes			Yes
Q9UIW2	PLXNA1	Yes		Yes	Yes	Yes		
Q96CN7	ISOC1		Yes					
Q8N2S1	LTBP4	Yes						
P61086	UBE2K		Yes					
Q8WUJ3	CEMIP	Yes						
P54802	NAGLU	Yes			Yes			
P16870	CPE	Yes						Yes
Q14839	CHD4							Yes
P84098	RPL19				Yes			
P25705	ATP5A1							Yes
O00303	EIF3F		Yes					
Q14011	CIRBP		Yes					Yes
Q32P28	LEPRE1	Yes			Yes			
P83110	HTRA3	Yes						
P62318	SNRPD3		Yes					
P24752	ACAT1							Yes
Q15417	CNN3		Yes					Yes
O15511	ARPC5		Yes					
O14773	TPP1	Yes			Yes			Yes

O15145	ARPC3		Yes					
P12272	PTHLH	Yes					Yes	
P09661	SNRPA1		Yes					
O75828	CBR3		Yes					Yes
P30566	ADSL							Yes
Q12907	LMAN2	Yes		Yes	Yes	Yes		
P04080	CSTB							Yes
P17812	CTPS1							Yes
P41567	EIF1		Yes					
P21741	MDK	Yes					Yes	
P17900	GM2A	Yes						
P54764	EPHA4	Yes		Yes	Yes	Yes		
P27824	CANX	Yes		Yes	Yes			Yes
Q96C86	DCPS		Yes					
Q9P0L0	VAPA			Yes				
P46782	RPS5		Yes					
P06493	CDK1							Yes
P50897	PPT1	Yes			Yes			
Q9BWS9	CHID1	Yes						
Q8NHP8	PLBD2	Yes		Yes	Yes			
P00918	CA2							Yes
P02458	COL2A1	Yes			Yes		Yes	Yes

O60869	EDF1		Yes					
Q9H773	DCTPP1		Yes					
P34059	GALNS	Yes			Yes			
Q15847	ADIRF		Yes					
Q53GS9	USP39		Yes					
Q8N1G4	LRRC47		Yes					
P51148	RAB5C		Yes		Yes			
Q86SQ4	GPR126	Yes		Yes	Yes	Yes		
Q9HBR0	SLC38A10		Yes	Yes				
Q9Y6E0	STK24		Yes					
P07741	APRT							Yes
P49767	VEGFC	Yes			Yes		Yes	
Q15651	HMGN3		Yes					
Q9H4F8	SMOC1	Yes						
P98172	EFNB1	Yes		Yes	Yes	Yes	Yes	
P28300	LOX	Yes			Yes			
O95881	TXNDC12	Yes						
Q9Y287	ITM2B		Yes	Yes	Yes	Yes		
O75390	CS							Yes
Q8TBC4	UBA3		Yes					
P01036	CST4	Yes						Yes
P68400	CSNK2A1		Yes					Yes

P62244	RPS15A		Yes		Yes			
Q99798	ACO2							Yes
Q16394	EXT1			Yes				
O95407	TNFRSF6B	Yes						
Q13541	EIF4EBP1		Yes					
P62263	RPS14		Yes					
Q03405	PLAUR	Yes			Yes	Yes		
P33240	CSTF2							Yes
Q9BQT9	CLSTN3	Yes		Yes		Yes		
O95302	FKBP9	Yes			Yes			
Q08431	MFGE8	Yes			Yes		Yes	
P04632	CAPNS1		Yes		Yes			Yes
Q6YP21	CCBL2		Yes					
P18077	RPL35A		Yes		Yes			
P42830	CXCL5	Yes					Yes	
P02750	LRG1	Yes						
P30626	SRI		Yes					
O00462	MANBA	Yes						
Q99574	SERPINI1	Yes			Yes			
Q13219	PAPPA	Yes						
P29323	EPHB2	Yes		Yes	Yes	Yes		
Q9UKU9	ANGPTL2	Yes			Yes		Yes	

Q14192	FHL2		Yes					
P09497	CLTB		Yes					Yes
P98088	MUC5AC	Yes						
Q99816	TSG101		Yes					
Q92890	UFD1L		Yes					
Q6ZRP7	QSOX2			Yes	Yes	Yes		
P05362	ICAM1	Yes		Yes	Yes	Yes	Yes	
Q9BXS5	AP1M1		Yes					
Q9UHL4	DPP7	Yes			Yes			
Q9NPR2	SEMA4B	Yes		Yes	Yes	Yes	Yes	
P31146	CORO1A		Yes					
Q9NRN5	OLFML3	Yes			Yes			
P54687	BCAT1							Yes
P21291	CSRP1							Yes
P50895	BCAM	Yes		Yes	Yes	Yes		
Q9Y3B8	REXO2		Yes					
Q12792	TWF1		Yes					
Q99439	CNN2							Yes
Q9NQP4	PFDN4		Yes					
P18827	SDC1	Yes		Yes		Yes		
Q07960	ARHGAP1							Yes
O43583	DENR		Yes					

P17516	AKR1C4							Yes
P48740	MASP1	Yes						
Q8IX30	SCUBE3	Yes						
P09603	CSF1	Yes		Yes	Yes	Yes	Yes	Yes
P50281	MMP14	Yes		Yes		Yes		
P30084	ECHS1		Yes					
P62847	RPS24		Yes					
Q9UBS4	DNAJB11	Yes		Yes				
O95967	EFEMP2	Yes					Yes	
ENST00000512123_NCI-H23_Mis:R1650Q	FRAS1	Yes		Yes	Yes	Yes		
Q969H8	MYDGF	Yes						
Q9Y2B0	CNPY2	Yes						
P02771	AFP	Yes						Yes
O60687	SRPX2	Yes						
P62277	RPS13		Yes					
Q15435	PPP1R7		Yes					
P42785	PRCP	Yes						
O95861	BPNT1		Yes					
P36957	DLST		Yes					
P61970	NUTF2		Yes					
P61224	RAP1B		Yes					

Q9HAT2	SIAE	Yes						
P23193	TCEA1		Yes					
P55265	ADAR							Yes
P18085	ARF4		Yes					Yes
P30043	BLVRB		Yes					Yes
Q9BQ67	GRWD1		Yes					
Q99426	TBCB		Yes					Yes
P62910	RPL32				Yes			
P32969	RPL9				Yes			
Q96IZ0	PAWR		Yes					
P62841	RPS15		Yes					
P09238	MMP10	Yes						
P55290	CDH13	Yes			Yes	Yes		Yes
P49411	TUFM		Yes					
P49862	KLK7	Yes						
P09496	CLTA		Yes					Yes
P08582	MFI2	Yes			Yes	Yes	Yes	
P55854	SUMO3		Yes					
P01040	CSTA							Yes
P49458	SRP9		Yes					
P59998	ARPC4		Yes					
P01889	HLA-B	Yes		Yes	Yes	Yes	Yes	

Q9GZT8	NIF3L1		Yes					
P21926	CD9			Yes		Yes		Yes
P37235	HPCAL1		Yes					
P12429	ANXA3							Yes
P02794	FTH1		Yes					
P62304	SNRPE		Yes					
P15151	PVR	Yes		Yes	Yes	Yes		
P36507	MAP2K2		Yes					
Q7Z2W4	ZC3HAV1				Yes			
P84085	ARF5		Yes					Yes
P45973	CBX5		Yes					
Q13085	ACACA							Yes
Q15185	PTGES3		Yes					
P61081	UBE2M		Yes					
Q9GZP0	PDGFD	Yes					Yes	
Q6P4A8	PLBD1	Yes			Yes			
P15291	B4GALT1		Yes	Yes				
Q9P2M7	CGN				Yes		Yes	
Q14703	MBTPS1	Yes		Yes				
P13674	P4HA1	Yes			Yes			
P46776	RPL27A		Yes		Yes			
Q9Y3A5	SBDS		Yes					

P07225	PROS1	Yes					Yes	
O43768	ENSA		Yes					
Q9UMX5	NENF	Yes						
P55196	MLLT4						Yes	
Q9NRA1	PDGFC	Yes					Yes	
P61812	TGFB2	Yes		Yes			Yes	
Q86UD1	OAF	Yes						
Q93052	LPP		Yes					
P61586	RHOA		Yes					Yes
Q9UKK3	PARP4							Yes
P06756	ITGAV	Yes		Yes	Yes	Yes		
P00568	AK1		Yes					Yes
Q9NX55	HYPK		Yes					
P54105	CLNS1A							Yes
Q9UBQ5	EIF3K		Yes					
P04899	GNAI2		Yes				Yes	
Q24JP5	TMEM132A	Yes		Yes	Yes	Yes		
P55145	MANF	Yes						
Q9NRV9	HEBP1						Yes	
Q12996	CSTF3							Yes
Q9BPX5	ARPC5L		Yes					
P55285	CDH6	Yes		Yes	Yes	Yes		Yes

Q9Y624	F11R	Yes		Yes	Yes	Yes		
O95292	VAPB			Yes				
ENST00000539941_LIM2099_Mis:N8S	COPS3		Yes					
P07478	PRSS2	Yes						
Q99961	SH3GL1		Yes					
Q99848	EBNA1BP2		Yes					
Q15758	SLC1A5		Yes	Yes	Yes	Yes		
P48745	NOV	Yes					Yes	
P12532	CKMT1A							Yes
P35268	RPL22		Yes					
P01037	CST1	Yes						
Q99988	GDF15	Yes			Yes			
P51572	BCAP31			Yes				
Q86SR1	GALNT10			Yes				
Q9BZM5	ULBP2	Yes		Yes	Yes	Yes	Yes	
Q13217	DNAJC3	Yes						
P35613	BSG	Yes		Yes	Yes	Yes		Yes
P36551	CPOX		Yes					
P60903	S100A10			Yes				
P49207	RPL34		Yes					
Q9BS26	ERP44	Yes						
P60953	CDC42		Yes					Yes

P84090	ERH		Yes					
P08579	SNRPB2		Yes					
P09525	ANXA4							Yes
O75718	CRTAP	Yes			Yes			
Q92673	SORL1	Yes		Yes	Yes	Yes		
Q9GZP4	PITHD1		Yes					
Q9UMY4	SNX12		Yes					
Q13438	OS9	Yes		Yes				
P29373	CRABP2							Yes
O14792	HS3ST1	Yes						
P41208	CETN2		Yes					Yes
O75940	SMNDC1		Yes					
Q14393	GAS6	Yes					Yes	
P52758	HRSP12		Yes					
O95834	EML2		Yes					
Q9Y5X9	LIPG	Yes			Yes			
P78536	ADAM17	Yes		Yes	Yes	Yes	Yes	
P50995	ANXA11		Yes					Yes
Q8IXL6	FAM20C	Yes		Yes				
P78406	RAE1		Yes					
P61225	RAP2B		Yes					
Q5JWF2	GNAS						Yes	

P20061	TCN1	Yes						
P31937	HIBADH		Yes					
Q8IVM0	CCDC50		Yes					
Q9UNW1	MINPP1	Yes			Yes			
Q9UJ70	NAGK		Yes					
Q86SF2	GALNT7			Yes				
P12277	CKB							Yes
P14854	COX6B1							Yes
Q96PD2	DCBLD2	Yes		Yes	Yes	Yes		
P49788	RARRES1	Yes						
Q92859	NEO1	Yes		Yes	Yes	Yes		
P14649	MYL6B		Yes		Yes			
P0DN79			Yes					
P10606	COX5B		Yes					Yes
P24941	CDK2							Yes
Q9Y237	PIN4		Yes					
Q9BUD6	SPON2	Yes					Yes	
O00300	TNFRSF11B	Yes			Yes			
O60925	PFDN1		Yes					
P98095	FBLN2	Yes			Yes		Yes	
P33908	MAN1A1			Yes				
P41227	NAA10		Yes					

P42766	RPL35		Yes		Yes			
Q9UHY7	ENOPH1		Yes					
Q03135	CAV1			Yes				Yes
Q6PCB0	VWA1	Yes						
O00592	PODXL	Yes		Yes	Yes	Yes	Yes	
Q9UBX5	FBLN5	Yes						
Q9UHG2	PCSK1N	Yes						
Q99614	TTC1		Yes					
Q04323	UBXN1		Yes					
Q8N114	SHISA5	Yes		Yes				
P26006	ITGA3	Yes		Yes	Yes	Yes		
Q8NBJ5	COLGALT1	Yes			Yes			
P98179	RBM3		Yes					
Q9HC84	MUC5B	Yes						
Q53FA7	TP53I3		Yes					
O14908	GIPC1		Yes					
Q969P0	IGSF8	Yes		Yes	Yes	Yes		
Q9NX58	LYAR		Yes					
Q14116	IL18		Yes				Yes	
P08174	CD55	Yes			Yes	Yes	Yes	Yes
P04085	PDGFA	Yes					Yes	
Q9NP97	DYNLRB1		Yes					

P08134	RHOC		Yes					Yes
Q13642	FHL1		Yes					
P41236	PPP1R2		Yes					
Q01433	AMPD2							Yes
Q9UIQ6	LNPEP		Yes	Yes	Yes	Yes		
Q9Y2A9	B3GNT3			Yes				
P26232	CTNNA2							Yes
P30519	HMOX2			Yes				
P63279	UBE2I		Yes					
Q4VC31	CCDC58		Yes					
O75396	SEC22B			Yes	Yes			
P01344	IGF2	Yes					Yes	
O60565	GREM1	Yes			Yes		Yes	
Q6FI81	CIAPIN1		Yes					
Q9H5V8	CDCP1	Yes		Yes	Yes	Yes		
P52799	EFNB2	Yes		Yes	Yes	Yes	Yes	
O95816	BAG2		Yes					
Q15121	PEA15		Yes					
Q12972	PPP1R8		Yes					
Q9BQA1	WDR77		Yes					
Q6P179	ERAP2				Yes			
P16278	GLB1	Yes			Yes			

O60828	PQBP1		Yes					
P19784	CSNK2A2							Yes
P09341	CXCL1	Yes					Yes	
P24666	ACP1		Yes					Yes
O14561	NDUFAB1		Yes					
P05091	ALDH2		Yes					Yes
P04004	VTN	Yes			Yes		Yes	
P09228	CST2	Yes						
P03973	SLPI	Yes					Yes	
P26572	MGAT1			Yes				
P35573	AGL							Yes
P60983	GMFB		Yes					
O00233	PSMD9		Yes					
P05156	CFI	Yes						
O75509	TNFRSF21	Yes		Yes	Yes	Yes		
P43121	MCAM	Yes		Yes	Yes	Yes		
Q92466	DDB2							Yes
P05060	CHGB	Yes						
Q02413	DSG1	Yes				Yes		
Q9BY76	ANGPTL4	Yes			Yes		Yes	
Q8IZ83	ALDH16A1		Yes					
O43681	ASNA1							Yes

Q9UKM7	MAN1B1		Yes	Yes				
P13987	CD59	Yes			Yes	Yes		Yes
P23634	ATP2B4			Yes		Yes		Yes
P78504	JAG1	Yes		Yes	Yes	Yes	Yes	Yes
O00479	HMGN4		Yes					
P20340	RAB6A		Yes					
Q7Z422	SZRD1		Yes					
Q05519	SRSF11		Yes					
P11279	LAMP1	Yes		Yes	Yes	Yes		
P09543	CNP							Yes
P40855	PEX19		Yes					
Q8IWS0	PHF6		Yes					
P15514	AREG	Yes		Yes	Yes	Yes	Yes	Yes
Q5TEC6	HIST2H3PS2	Yes						
O15382	BCAT2							Yes
P84243	H3F3A		Yes					
O15514	POLR2D		Yes					
Q14512	FGFBP1	Yes						
P55957	BID		Yes					Yes
Q04721	NOTCH2	Yes		Yes	Yes	Yes		
Q7RTV0	PHF5A		Yes					
O75340	PDCD6		Yes					

Q6EMK4	VASN	Yes		Yes	Yes	Yes		
P35318	ADM	Yes					Yes	Yes
O94826	TOMM70A			Yes				
O00115	DNASE2	Yes						
Q15785	TOMM34		Yes					
Q6EEV6	SUMO4		Yes					
P16435	POR			Yes				
Q9UNN8	PROCR	Yes		Yes	Yes	Yes		
Q9H488	POFUT1	Yes						
Q13510	ASAH1	Yes			Yes			Yes
P04156	PRNP	Yes		Yes	Yes	Yes		
Q96FQ6	S100A16		Yes					
P09417	QDPR						Yes	
Q8NBZ7	UXS1			Yes				
Q9Y2E5	MAN2B2	Yes			Yes			
O15400	STX7			Yes				
P32929	CTH							Yes
P53004	BLVRA							Yes
P61020	RAB5B		Yes					
Q14137	BOP1		Yes					
P04066	FUCA1	Yes						
P0DN86		Yes						

Q05707	COL14A1	Yes		Yes	Yes		Yes	
P18615	NELFE		Yes					
O00244	ATOX1							Yes
P08236	GUSB	Yes		Yes	Yes			
O00161	SNAP23		Yes					
Q14508	WFDC2	Yes		Yes				
Q9H098	FAM107B		Yes					
P83881	RPL36A		Yes					
O14562	UBFD1		Yes					
P10301	RRAS		Yes					
Q13526	PIN1		Yes					
Q8NCC3	PLA2G15	Yes						
P22466	GAL	Yes		Yes			Yes	
Q92692	PVRL2	Yes		Yes	Yes	Yes		
Q86SJ2	AMIGO2	Yes		Yes	Yes	Yes		
Q9NVS9	PNPO		Yes					
P13598	ICAM2	Yes		Yes	Yes	Yes	Yes	
Q5T013	HYI		Yes					
P60866	RPS20		Yes					
P26885	FKBP2	Yes						
P18859	ATP5J							Yes
Q68BL7	OLFML2A	Yes			Yes			

O43505	B4GAT1			Yes	Yes			
Q9BW91	NUDT9	Yes						
P07311	ACYP1							Yes
P62633	CNBP		Yes					
Q9ULZ3	PYCARD		Yes					
Q08345	DDR1	Yes		Yes		Yes		Yes
P27658	COL8A1	Yes					Yes	Yes
P21964	COMT			Yes				Yes
Q9HB63	NTN4	Yes		Yes			Yes	
P63173	RPL38		Yes					
P50402	EMD			Yes				
Q13444	ADAM15	Yes		Yes	Yes	Yes	Yes	
P02654	APOC1	Yes						Yes
P15529	CD46	Yes		Yes	Yes	Yes		
P51452	DUSP3		Yes					
P22304	IDS	Yes						
P08833	IGFBP1	Yes						
Q86X55	CARM1		Yes					
Q5JTV8	TOR1AIP1				Yes			
O95965	ITGBL1	Yes						
Q9UJJ9	GNPTG	Yes			Yes			
Q10469	MGAT2	Yes		Yes				

Q15223	PVRL1	Yes		Yes	Yes	Yes		
O95994	AGR2	Yes						
Q9Y3A3	MOB4		Yes					
Q9UBQ6	EXTL2			Yes	Yes			
Q14194	CRMP1							Yes
P55287	CDH11	Yes		Yes	Yes	Yes		Yes
P00734	F2	Yes			Yes		Yes	
P20339	RAB5A		Yes					
P58397	ADAMTS12	Yes						
Q12797	ASPH		Yes	Yes	Yes			Yes
Q9Y6C2	EMILIN1	Yes			Yes			
Q9GZX9	TWSG1	Yes						
O60232	SSSCA1		Yes					
Q5JRA6	MIA3	Yes			Yes			
Q9UBG0	MRC2	Yes		Yes	Yes	Yes		
Q9Y653	GPR56	Yes		Yes	Yes	Yes		
P21127	CDK11B							Yes
Q8N0V5	GCNT2			Yes				
P61966	AP1S1							Yes
Q14114	LRP8	Yes		Yes		Yes		
Q8IWU5	SULF2	Yes		Yes	Yes			
Q9P2B2	PTGFRN	Yes		Yes	Yes	Yes		

P35270	SPR		Yes					
Q9H173	SIL1	Yes			Yes			
Q8NFL0	B3GNT7	Yes		Yes				
P27105	STOM			Yes				
O43395	PRPF3		Yes					
P11441	UBL4A		Yes					
Q12913	PTPRJ	Yes		Yes	Yes	Yes		
Q9Y263	PLAA		Yes					
Q9UIJ7	AK3		Yes					
Q8WWY3	PRPF31		Yes					
Q9UI30	TRMT112		Yes					
Q13557	CAMK2D							Yes
P56211	ARPP19		Yes					
P51688	SGSH	Yes						
O75976	CPD	Yes		Yes	Yes	Yes		Yes
Q9NVD7	PARVA		Yes					
Q9Y2V2	CARHSP1		Yes					
P04183	TK1		Yes					
Q9BYG3	NIFK		Yes					
Q9UEY8	ADD3							Yes
O75351	VPS4B		Yes					
P55735	SEC13		Yes					

P60033	CD81			Yes				Yes
P05452	CLEC3B	Yes						
P05062	ALDOB							Yes
P04114	APOB	Yes			Yes		Yes	Yes
P02795	MT2A		Yes					
P31327	CPS1							Yes
Q13591	SEMA5A	Yes		Yes	Yes	Yes	Yes	
P09758	TACSTD2	Yes		Yes	Yes	Yes		
Q9Y5A9	YTHDF2		Yes					
Q13610	PWP1		Yes					
Q9H3K6	BOLA2		Yes					
Q9NR09	BIRC6				Yes			
O60513	B4GALT4	Yes		Yes				
O00762	UBE2C		Yes					
P30837	ALDH1B1		Yes					Yes
P35611	ADD1							Yes
Q969Q0	RPL36AL		Yes					
P54760	EPHB4	Yes		Yes	Yes	Yes		
Q9UBI6	GNG12		Yes					
Q9BSJ8	ESYT1			Yes				
Q9HB40	SCPEP1	Yes						
Q96S86	HAPLN3	Yes						

Q14696	MESDC2	Yes						
P30533	LRPAP1	Yes		Yes	Yes		Yes	
Q9BPZ3	PAIP2		Yes					
O15305	PMM2		Yes					
Q6P988	NOTUM	Yes						
P49257	LMAN1	Yes		Yes				
Q03167	TGFBR3	Yes		Yes	Yes	Yes		
Q96IU4	ABHD14B		Yes					
Q99650	OSMR	Yes		Yes	Yes	Yes		
Q9NP84	TNFRSF12A	Yes		Yes				
ENST00000379086_NCI-H23_Mis:R399H	P4HA2	Yes			Yes			
Q00765	REEP5			Yes				
O95721	SNAP29		Yes					
P21283	ATP6V1C1							Yes
Q05048	CSTF1		Yes					Yes
O14657	TOR1B	Yes		Yes	Yes			
Q13895	BYSL		Yes					
P25325	MPST		Yes					
O94804	STK10		Yes					
Q9UDY4	DNAJB4		Yes					
O43324	EEF1E1		Yes					
P09601	HMOX1			Yes				

O94766	B3GAT3			Yes				
P62070	RRAS2		Yes					
Q6UX04	CWC27		Yes					
O94813	SLIT2	Yes			Yes		Yes	
P40189	IL6ST	Yes		Yes	Yes	Yes		
O75934	BCAS2		Yes					
Q9Y5L4	TIMM13		Yes					
P13747	HLA-E	Yes		Yes	Yes	Yes	Yes	
Q15836	VAMP3			Yes				
Q15599	SLC9A3R2		Yes					
Q8WZA0	LZIC		Yes					
Q9H1B5	XYLT2			Yes				
P63165	SUMO1		Yes					
O43291	SPINT2	Yes		Yes		Yes		
Q15198	PDGFRL	Yes						
Q9UM22	EPDR1			Yes	Yes			
P13284	IFI30	Yes			Yes			
P24347	MMP11	Yes						
Q96C90	PPP1R14B		Yes					
P98066	TNFAIP6	Yes						
P20073	ANXA7		Yes					Yes
Q9GZZ9	UBA5			Yes				

P48729	CSNK1A1							Yes
Q6GMV3	PTRHD1		Yes					
P17050	NAGA	Yes			Yes			
P52823	STC1	Yes						
P41271	NBL1	Yes						
P05161	ISG15		Yes					
Q9Y3B9	RRP15		Yes					
P49407	ARRB1							Yes
P62837	UBE2D2		Yes					
P81605	DCD	Yes						
Q96CG8	CTHRC1	Yes					Yes	
Q01459	CTBS	Yes						
Q8NBF2	NHLRC2		Yes					
Q9BQ61	C19orf43		Yes					
P05155	SERPING1	Yes			Yes		Yes	Yes
P19438	TNFRSF1A	Yes		Yes	Yes	Yes		
P49748	ACADVL							Yes
Q8NBJ7	SUMF2	Yes						
Q9C0B5	ZDHHC5			Yes	Yes	Yes		
Q9Y3L5	RAP2C		Yes					
Q9Y5J7	TIMM9		Yes					
P62072	TIMM10		Yes					

P25774	CTSS	Yes						Yes
P47914	RPL29		Yes					
P06730	EIF4E		Yes					
Q07812	BAX		Yes	Yes				Yes
Q14790	CASP8							Yes
P49755	TMED10	Yes		Yes				
O43488	AKR7A2		Yes					
P49459	UBE2A		Yes					
O60220	TIMM8A		Yes					
Q7Z4H8	KDELC2	Yes			Yes			
Q9Y6N7	ROBO1	Yes			Yes	Yes		
P55011	SLC12A2		Yes	Yes	Yes	Yes		
Q8N5I2	ARRDC1		Yes					
Q9UKF6	CPSF3		Yes					
P20020	ATP2B1			Yes				Yes
Q13938	CAPS		Yes					Yes
P17813	ENG	Yes		Yes	Yes	Yes		
Q9Y316	MEMO1		Yes					
Q8TB73	NDNF	Yes						
Q3MHD2	LSM12		Yes					
P56545	CTBP2							Yes
Q00534	CDK6							Yes

Q14574	DSC3	Yes		Yes		Yes		
Q96ER3	SAAL1		Yes					
P15927	RPA2		Yes					
P42126	ECI1		Yes					Yes
P25391	LAMA1	Yes			Yes		Yes	
P32321	DCTD		Yes					Yes
Q96C01	FAM136A		Yes					
P20674	COX5A		Yes					
P01111	NRAS		Yes					
P15153	RAC2		Yes					
Q92785	DPF2		Yes					
O14828	SCAMP3			Yes				
P07919	UQCRH		Yes					
Q96J84	KIRREL	Yes		Yes	Yes	Yes		
P37840	SNCA		Yes					
O00264	PGRMC1			Yes				
P67870	CSNK2B		Yes					Yes
Q8WUD1	RAB2B		Yes					
P20062	TCN2	Yes					Yes	
P18031	PTPN1			Yes				
P48507	GCLM		Yes					
Q13951	CBFB							Yes

Q9BY32	ITPA		Yes					
O75503	CLN5		Yes	Yes	Yes			Yes
Q9BXK5	BCL2L13			Yes				
Q9UN70	PCDHGC3			Yes	Yes	Yes		
Q14684	RRP1B		Yes					
Q96DI7	SNRNP40		Yes					
P13073	COX4I1			Yes				Yes
P16220	CREB1							Yes
Q9Y294	ASF1A		Yes					
O95633	FSTL3	Yes						
Q16775	HAGH		Yes					
Q8WXA9	SREK1		Yes					
Q96EU7	C1GALT1C1			Yes				
Q9NUP9	LIN7C						Yes	
Q86WQ0	NR2C2AP		Yes					
P20827	EFNA1	Yes			Yes	Yes	Yes	
Q9Y2S6	TMA7		Yes					
P78324	SIRPA	Yes		Yes	Yes	Yes		
Q6ZMP0	THSD4	Yes			Yes			
Q7L9L4	MOB1B		Yes					
O75380	NDUFS6		Yes					
P61927	RPL37		Yes					

Q8N543	OGFOD1				Yes			
O75629	CREG1	Yes			Yes			
P39880	CUX1							Yes
P09529	INHBB	Yes					Yes	
P34096	RNASE4	Yes		Yes				
O43464	HTRA2		Yes	Yes				
Q8WUW1	BRK1		Yes					
Q9UKL0	RCOR1		Yes					
Q5TDH0	DDI2		Yes					
P52943	CRIP2		Yes					Yes
P61077	UBE2D3		Yes					
Q99417	MYCBP		Yes					
Q99941	ATF6B		Yes		Yes			Yes
O00442	RTCA		Yes					
P05026	ATP1B1			Yes	Yes	Yes		Yes
Q96AT1	KIAA1143		Yes					
P20933	AGA	Yes						Yes
P61006	RAB8A		Yes					
Q16769	QPCT	Yes						
P78310	CXADR	Yes		Yes	Yes	Yes		Yes
Q9HCU0	CD248	Yes		Yes		Yes		
P51153	RAB13		Yes					

Q01650	SLC7A5		Yes	Yes	Yes	Yes		
P14635	CCNB1							Yes
Q92917	GPKOW		Yes					
Q14061	COX17		Yes					
P15289	ARSA	Yes						Yes
Q12849	GRSF1		Yes					
P13473	LAMP2	Yes		Yes	Yes	Yes		
P54709	ATP1B3		Yes	Yes	Yes	Yes		Yes
P17275	JUNB		Yes					
O75348	ATP6V1G1		Yes					
Q96M27	PRRC1		Yes					
Q13555	CAMK2G		Yes					Yes
P17535	JUND		Yes					
Q9UN81	L1RE1		Yes					
P10109	FDX1		Yes					
Q641Q3	METRNL	Yes						
Q06033	ITIH3	Yes						
P36405	ARL3		Yes					Yes
Q99622	C12orf57		Yes					
P46100	ATRX				Yes			Yes
Q9H0C8	ILKAP		Yes					
P15941	MUC1	Yes		Yes		Yes		

P25940	COL5A3	Yes			Yes		Yes	
O95372	LYPLA2		Yes					
Q16539	MAPK14							Yes
Q9Y3C1	NOP16		Yes					
O60512	B4GALT3			Yes	Yes			
P08493	MGP	Yes						
P12644	BMP4	Yes					Yes	Yes
Q86Y82	STX12			Yes				
Q8IV08	PLD3		Yes	Yes	Yes			
Q10472	GALNT1			Yes	Yes			
Q96TC7	RMDN3			Yes				
O14618	CCS		Yes					
P01116	KRAS		Yes					
P13984	GTF2F2		Yes					
Q9BV40	VAMP8			Yes				
Q14739	LBR		Yes	Yes				
P62861	FAU		Yes					
Q13363	CTBP1							Yes
Q9NVM1	EVA1B			Yes				
Q9NQ48	LZTFL1		Yes		Yes			
Q08397	LOXL1	Yes						
O00422	SAP18		Yes					

Q9P016	THYN1		Yes					
A8MXV4	NUDT19		Yes					
P09958	FURIN	Yes		Yes		Yes		
P02788	LTF	Yes			Yes		Yes	
P54886	ALDH18A1		Yes					
P41240	CSK							Yes
P00742	F10	Yes					Yes	
Q9ULF5	SLC39A10	Yes		Yes	Yes	Yes		
Q8TDW7	FAT3	Yes		Yes	Yes	Yes		
Q99519	NEU1			Yes	Yes			
Q9Y5P6	GMPPB		Yes		Yes			
P13500	CCL2	Yes					Yes	
P54803	GALC	Yes						
P11362	FGFR1	Yes		Yes	Yes	Yes		
Q13405	MRPL49		Yes					Yes
Q13685	AAMP		Yes					Yes
Q7Z304	MAMDC2	Yes						
Q96JY6	PDLIM2		Yes					
P61513	RPL37A		Yes		Yes			
Q07065	CKAP4		Yes	Yes				
O95486	SEC24A		Yes					
Q01638	IL1RL1	Yes		Yes		Yes		

Q8NI22	MCFD2	Yes						
P43235	CTSK	Yes			Yes			Yes
Q9BQ16	SPOCK3	Yes						
Q14554	PDIA5	Yes						
Q6UXD5	SEZ6L2	Yes		Yes	Yes	Yes		
P18846	ATF1							Yes
Q86VZ4	LRP11	Yes		Yes	Yes	Yes		
Q16625	OCLN			Yes				
P50238	CRIP1							Yes
P30047	GCHFR		Yes					
P55283	CDH4	Yes		Yes	Yes	Yes		Yes
Q6UXH9	PAMR1	Yes						
P36404	ARL2							Yes
Q9Y6A4	CFAP20		Yes					
P36543	ATP6V1E1							Yes
Q9Y244	POMP		Yes					
P48960	CD97	Yes		Yes	Yes	Yes		Yes
Q9UEW8	STK39		Yes					
P41223	BUD31		Yes					
Q8WX77	IGFBPL1	Yes						
P62330	ARF6		Yes					Yes
Q6FIF0	ZFAND6		Yes					

P04920	SLC4A2			Yes	Yes			
P00167	CYB5A			Yes				Yes
Q9UMS0	NFU1		Yes					
Q9H0S4	DDX47		Yes					
Q8TEA8	DTD1		Yes					
P41222	PTGDS	Yes						
P04843	RPN1	Yes		Yes	Yes	Yes		
Q16527	CSRP2		Yes					Yes
Q9BY42	RTFDC1		Yes					
P24386	CHM							Yes
Q7L5D6	GET4		Yes					
P30260	CDC27							Yes
Q8NFH5	NUP35		Yes					
O00461	GOLIM4			Yes	Yes			
P27707	DCK							Yes
Q6BCY4	CYB5R2		Yes					
P42892	ECE1		Yes	Yes	Yes	Yes		
O00241	SIRPB1	Yes		Yes	Yes	Yes		
Q6P4E1	CASC4			Yes	Yes			
P83876	TXNL4A		Yes					
Q13253	NOG	Yes						
P51965	UBE2E1		Yes					

Q9H8J5	MANSC1	Yes		Yes		Yes		
Q9UHN6	TMEM2			Yes	Yes			
P19971	TYMP		Yes					
P12107	COL11A1	Yes			Yes		Yes	Yes
L0R819	ASNSD1		Yes					
P50851	LRBA							Yes
Q9C0A0	CNTNAP4	Yes		Yes		Yes		
Q00888	PSG4	Yes						
Q92575	UBXN4		Yes					
P48735	IDH2				Yes			
Q9UQ53	MGAT4B			Yes	Yes			
P13716	ALAD		Yes					Yes
Q9BRK3	MXRA8	Yes		Yes	Yes	Yes		
P29400	COL4A5	Yes					Yes	Yes
Q9GZN4	PRSS22	Yes						
Q9Y5J9	TIMM8B		Yes					
Q9NS68	TNFRSF19	Yes		Yes		Yes		
Q13426	XRCC4		Yes					
Q9Y547	HSPB11		Yes					
Q9GZM5	YIPF3		Yes	Yes				
Q9UNE7	STUB1		Yes					
Q9C0C4	SEMA4C	Yes		Yes	Yes	Yes	Yes	

P03950	ANG	Yes		Yes				Yes
Q9Y314	NOSIP		Yes					
Q9H9T3	ELP3		Yes					
Q9NY97	B3GNT2				Yes			
Q49A17	GALNTL6			Yes				
Q9H477	RBKS		Yes					
Q8N668	COMMD1		Yes					
P82909	MRPS36		Yes					
O43715	TRIAP1		Yes					
Q13643	FHL3		Yes					
P47972	NPTX2	Yes			Yes			
O95989	NUDT3		Yes					
Q13214	SEMA3B	Yes		Yes			Yes	
Q9H7Z7	PTGES2		Yes					
Q96HY6	DDRGK1			Yes				
Q9H9K5	ERVMER34-1	Yes		Yes		Yes		
Q9NUG6	PDRG1		Yes					
Q9NPH3	IL1RAP	Yes		Yes	Yes	Yes		
Q6UXH1	CRELD2	Yes			Yes			
P21246	PTN	Yes		Yes			Yes	
Q9BXI9	C1QTNF6		Yes	Yes				
P40199	CEACAM6	Yes			Yes	Yes		

Q9UKP4	ADAMTS7	Yes						
Q9H814	PHAX		Yes					
Q9NWM8	FKBP14	Yes			Yes			
Q9BVJ7	DUSP23		Yes					
Q8NFJ5	GPRC5A			Yes	Yes	Yes		
Q12979	ABR							Yes
Q92572	AP3S1							Yes
Q9C005	DPY30		Yes					
Q9BRT3	MIEN1		Yes					
P30049	ATP5D		Yes					Yes
Q9NR33	POLE4		Yes					
Q11128	FUT5			Yes				
Q9Y2U8	LEMD3			Yes				
P58215	LOXL3	Yes						
Q6DD88	ATL3		Yes	Yes				
Q96AJ9	VTI1A			Yes				
Q8N474	SFRP1	Yes		Yes			Yes	
O75197	LRP5	Yes		Yes	Yes	Yes		
Q9BQI0	AIF1L		Yes					
Q9HCY8	S100A14		Yes					
O43665	RGS10		Yes					
O95297	MPZL1	Yes		Yes	Yes	Yes		

Q86W42	THOC6		Yes					
P63146	UBE2B		Yes					
Q9H9Q2	COPS7B		Yes					
O43286	B4GALT5			Yes	Yes			
Q96B54	ZNF428		Yes					
Q7LGC8	CHST3		Yes		Yes			
Q08722	CD47	Yes		Yes	Yes	Yes		Yes
Q9H0P0	NT5C3A			Yes				
Q02487	DSC2	Yes		Yes	Yes	Yes		
O15162	PLSCR1		Yes					
O60245	PCDH7	Yes		Yes	Yes	Yes		
Q7Z3B1	NEGR1	Yes			Yes	Yes		
Q8IXH7	NELFCD		Yes					
O15232	MATN3	Yes						
P06702	S100A9						Yes	
P51570	GALK1		Yes					
P06276	BCHE	Yes		Yes	Yes			Yes
P63027	VAMP2		Yes	Yes				
P84101	SERF2		Yes					
O14944	EREG	Yes		Yes		Yes	Yes	
Q9NTM9	CUTC		Yes					
P11166	SLC2A1			Yes	Yes	Yes		

Q6NW40	RGMB	Yes				Yes	Yes	
A6NIH7	UNC119B		Yes					
Q96QD8	SLC38A2			Yes		Yes		
Q8WUP2	FBLIM1		Yes					
Q9BWJ5	SF3B5		Yes					
O43570	CA12	Yes		Yes	Yes	Yes		Yes
O94923	GLCE			Yes	Yes			
O75063	FAM20B			Yes	Yes			
Q16644	MAPKAPK3		Yes					
Q8IXB1	DNAJC10			Yes				
O14907	TAX1BP3		Yes					
Q9NR99	MXRA5	Yes			Yes			
Q6ZNF0	PAPL	Yes						
P08294	SOD3	Yes						
Q7LG56	RRM2B			Yes				
P54756	EPHA5				Yes	Yes		
Q9UBV8	PEF1		Yes					
Q9NX62	IMPAD1			Yes	Yes			
O95502	NPTXR			Yes				
Q3ZCQ8	TIMM50		Yes					
Q92484	SMPDL3A	Yes						
Q10588	BST1	Yes		Yes	Yes	Yes	Yes	Yes

Q9Y3D0	FAM96B		Yes					
Q14165	MLEC	Yes		Yes				
O75496	GMNN		Yes					
O95571	ETHE1		Yes					
Q9Y680	FKBP7	Yes			Yes			
Q86VR8	FJX1			Yes				
Q8N6N3	C1orf52		Yes					
P08571	CD14	Yes			Yes	Yes	Yes	Yes
Q03403	TFF2	Yes						
Q96EE4	CCDC126			Yes	Yes			
Q14653	IRF3		Yes					
Q9UK22	FBXO2		Yes					
P48509	CD151		Yes	Yes	Yes	Yes		Yes
Q9BT09	CNPY3	Yes						
Q9BYN0	SRXN1		Yes					
Q15382	RHEB		Yes					
P13995	MTHFD2		Yes					
Q8N183	NDUFAF2		Yes					
P51911	CNN1							Yes
Q8TBA6	GOLGA5			Yes				
O75071	EFCAB14		Yes	Yes	Yes			
P30838	ALDH3A1		Yes					Yes

Q99547	MPHOSPH6		Yes					
P51511	MMP15	Yes		Yes				
P78423	CX3CL1	Yes		Yes		Yes	Yes	
Q96A33	CCDC47	Yes						
Q96NY8	PVRL4	Yes		Yes		Yes		
Q9NX40	OCIAD1		Yes					
P00326	ADH1C							Yes
P53985	SLC16A1			Yes		Yes		
P49356	FNTB		Yes					
Q96ME1	FBXL18		Yes					
Q8WUA8	TSKU	Yes						
P00995	SPINK1	Yes					Yes	
P01583	IL1A						Yes	
Q15238	PSG5	Yes						
O75354	ENTPD6			Yes				Yes
O95202	LETM1			Yes				
O95249	GOSR1		Yes	Yes				
Q03113	GNA12		Yes					
P50750	CDK9							Yes
Q9BZX2	UCK2		Yes					
P51397	DAP		Yes					Yes
P28908	TNFRSF8	Yes		Yes	Yes	Yes		Yes

P34949	MPI		Yes					
P57076	C21orf59		Yes					
Q6NZ67	MZT2B		Yes					
Q9Y2Q5	LAMTOR2		Yes					
Q9UMD9	COL17A1			Yes				Yes
P09110	ACAA1		Yes		Yes			Yes
P31751	AKT2							Yes
Q96FE7	PIK3IP1	Yes		Yes		Yes		
Q9NXR7	BRE		Yes					
Q9BTL3	FAM103A1		Yes					
O60488	ACSL4			Yes				
Q9HC97	GPR35			Yes		Yes		
Q99784	OLFM1				Yes			
P43363	MAGEA10		Yes					
Q7Z434	MAVS			Yes				
P20645	M6PR	Yes		Yes	Yes	Yes		
P05089	ARG1							Yes
P10145	CXCL8	Yes		Yes				
Q9GZN8	C20orf27		Yes					
Q9Y2S7	POLDIP2		Yes					
P61218	POLR2F		Yes					
P53611	RABGGTB		Yes					

Q9NP66	HMG20A		Yes					
O43752	STX6			Yes				
P51668	UBE2D1		Yes					
Q9NUM4	TMEM106B		Yes	Yes	Yes	Yes		
Q13822	ENPP2	Yes		Yes				
O76071	CIAO1		Yes					
Q92542	NCSTN	Yes		Yes	Yes	Yes		
Q8N130	SLC34A3			Yes		Yes		
Q9H3N1	TMX1	Yes		Yes				
Q96LR5	UBE2E2		Yes					
Q9NZP8	C1RL	Yes						
Q99523	SORT1	Yes		Yes	Yes	Yes		
Q8NBK3	SUMF1	Yes			Yes			
O43556	SGCE			Yes	Yes	Yes		
P07998	RNASE1	Yes		Yes				
P55001	MFAP2	Yes					Yes	
P50613	CDK7							Yes
P09466	PAEP	Yes						
Q96E11	MRRF		Yes					
Q68D85	NCR3LG1	Yes		Yes	Yes	Yes		
P53680	AP2S1							Yes
Q9GZT3	SLIRP		Yes					

P04731	MT1A		Yes					
P49641	MAN2A2			Yes	Yes			
P19256	CD58	Yes		Yes	Yes	Yes		Yes
Q92854	SEMA4D	Yes		Yes	Yes	Yes	Yes	
Q969T4	UBE2E3		Yes					
P36897	TGFBR1	Yes		Yes		Yes		
P04216	THY1	Yes			Yes	Yes		
Q8IUR7	ARMC8		Yes					
P22352	GPX3	Yes						
Q96BH1	RNF25		Yes					
Q8WUH1	CHURC1		Yes					
P84157	MXRA7			Yes				
P42574	CASP3							Yes
Q99828	CIB1		Yes					
Q96S66	CLCC1	Yes		Yes				
Q13158	FADD		Yes					
O75144	ICOSLG	Yes		Yes	Yes	Yes		
O75581	LRP6	Yes		Yes		Yes		
P18084	ITGB5	Yes		Yes	Yes	Yes		
Q9C0E2	XPO4		Yes					
Q9Y646	CPQ	Yes			Yes			
Q8WVN6	SECTM1	Yes		Yes		Yes		

Q96KG7	MEGF10	Yes		Yes		Yes		
Q92633	LPAR1			Yes		Yes		
P37198	NUP62		Yes					
P08648	ITGA5	Yes		Yes	Yes	Yes		
Q5JTJ3	COA6		Yes					
Q96JB6	LOXL4	Yes			Yes			
Q9HC07	TMEM165	Yes		Yes				
Q9Y5U2	TSSC4		Yes					
Q9BUN1	MENT	Yes						
P60604	UBE2G2		Yes					
P98082	DAB2							Yes
P63272	SUPT4H1		Yes					
Q9P2E7	PCDH10	Yes		Yes		Yes		
P0CG35	TMSB15B		Yes					
Q9UMR5	PPT2	Yes		Yes	Yes			
P22528	SPRR1B		Yes					
Q08554	DSC1	Yes		Yes		Yes		
Q9NPD8	UBE2T		Yes					
Q9H425	C1orf198		Yes					
P01127	PDGFB	Yes					Yes	
P61024	CKS1B							Yes
P20248	CCNA2							Yes

Q8NHZ8	CDC26		Yes					
Q9H497	TOR3A	Yes			Yes			
Q9BVC5	C2orf49		Yes					
P54710	FXYD2			Yes				Yes
Q13641	TPBG	Yes		Yes	Yes	Yes		
Q02297	NRG1			Yes		Yes	Yes	
O60476	MAN1A2		Yes	Yes				
P05187	ALPP	Yes			Yes	Yes		Yes
Q14331	FRG1		Yes					
O75191	XYLB		Yes					
Q6UY11	DLK2	Yes		Yes		Yes	Yes	
Q9H6X2	ANTXR1	Yes		Yes	Yes	Yes		
P54753	EPHB3	Yes		Yes	Yes	Yes		
Q9NRY6	PLSCR3		Yes					
Q11206	ST3GAL4			Yes	Yes			
Q29980	MICB	Yes			Yes	Yes		
P04844	RPN2	Yes		Yes	Yes			
P49454	CENPF							Yes
Q5JS54	PSMG4		Yes					
P19876	CXCL3	Yes					Yes	
Q9P035	HACD3			Yes				
P16615	ATP2A2		Yes	Yes				Yes

Q9BRX5	GINS3		Yes					
Q9C0H2	TTYH3			Yes	Yes	Yes		
Q9UJC5	SH3BGRL2		Yes					
Q9P0J7	KCMF1		Yes					
Q15389	ANGPT1	Yes			Yes		Yes	Yes
P14210	HGF	Yes					Yes	
P62253	UBE2G1		Yes					
Q7Z3B4	NUP54		Yes					
O60353	FZD6	Yes		Yes		Yes		
O43678	NDUFA2		Yes					
Q9Y508	RNF114		Yes					
P19957	PI3	Yes		Yes			Yes	
Q6UW78	UQCC3			Yes				
Q9Y3D6	FIS1			Yes				
P56181	NDUFV3		Yes					
Q00535	CDK5							Yes
P63218	GNG5		Yes					
O43709	WBSCR22		Yes					
P13501	CCL5	Yes					Yes	
Q9UBI1	COMMD3		Yes					
Q8IXQ4	GPALPP1		Yes					
P02656	APOC3	Yes					Yes	Yes

Q9NVG8	TBC1D13		Yes					
P50583	NUDT2		Yes					Yes
ENST00000354250_SW620_Mis:D277V	NDUFV3		Yes					
O14933	UBE2L6		Yes					
Q7Z5L7	PODN	Yes						
O43805	SSNA1		Yes					
O00220	TNFRSF10A			Yes		Yes		
Q15388	TOMM20			Yes				
P55210	CASP7				Yes			Yes
O95197	RTN3			Yes				
Q9P1F3	ABRACL		Yes					
Q8N441	FGFRL1	Yes		Yes	Yes	Yes		
P52803	EFNA5	Yes			Yes	Yes	Yes	
P84095	RHOG		Yes					Yes
Q8N128	FAM177A1		Yes					
O95479	H6PD	Yes						
P62745	RHOB		Yes					Yes
P98170	XIAP							Yes
P29558	RBMS1		Yes					
A8CG34	POM121C			Yes				
O43181	NDUFS4		Yes					
P05141	SLC25A5			Yes				Yes

Q15517	CDSN	Yes			Yes			
O96007	MOCS2		Yes					
Q9NSA3	CTNNBIP1		Yes					
P04626	ERBB2	Yes		Yes	Yes	Yes		
Q16651	PRSS8	Yes				Yes		
Q8TED0	UTP15		Yes					
Q5T2E6	C10orf76		Yes					
P22681	CBL							Yes
Q9GZV5	WWTR1		Yes					
Q9NZU0	FLRT3	Yes		Yes		Yes		
P07738	BPGM							Yes
Q07817	BCL2L1			Yes				Yes
Q9ULX3	NOB1		Yes					
Q8N2F6	ARMC10	Yes		Yes				
O75493	CA11	Yes						
Q8N4A0	GALNT4			Yes				
Q3B726	TWISTNB		Yes					
Q8WWX9	SELM	Yes		Yes				
Q9BXR0	QTRT1		Yes					
P05109	S100A8						Yes	
P60468	SEC61B		Yes	Yes				
Q8NFQ8	TOR1AIP2		Yes	Yes	Yes			

Q8NHG7	SVIP		Yes					
Q9NZ72	STMN3		Yes					
Q86VE9	SERINC5			Yes		Yes		
Q9BUR5	APOO			Yes				
O60259	KLK8	Yes						
Q53QV2	LBH		Yes					
P05231	IL6	Yes					Yes	
Q96Q45	TMEM237			Yes				
Q99969	RARRES2	Yes					Yes	
P25445	FAS	Yes			Yes	Yes		Yes
Q92597	NDRG1		Yes					
Q9NX63	CHCHD3		Yes					
Q9UMF0	ICAM5	Yes		Yes	Yes	Yes	Yes	
Q14520	HABP2	Yes						
P23511	NFYA		Yes					
P51809	VAMP7			Yes				
P36954	POLR2I		Yes					
Q9NPE3	NOP10		Yes					
P23468	PTPRD	Yes		Yes	Yes	Yes		
Q14139	UBE4A		Yes					
P17302	GJA1			Yes		Yes		
O43529	CHST10			Yes				

P01135	TGFA	Yes		Yes		Yes	Yes	
P01303	NPY	Yes		Yes			Yes	
Q9Y3Y2	CHTOP		Yes					
Q9Y3D8	AK6		Yes					
O15126	SCAMP1			Yes				
Q86WR0	CCDC25		Yes					
Q66PJ3	ARL6IP4		Yes					
Q99542	MMP19	Yes						
Q8N8Z6	DCBLD1	Yes		Yes	Yes	Yes		
Q15796	SMAD2		Yes					
P25208	NFYB		Yes					
Q9HBI1	PARVB		Yes					
Q9NZJ9	NUDT4		Yes					
Q8IZA0	KIAA0319L			Yes	Yes			
Q15413	RYR3			Yes	Yes			
P35443	THBS4	Yes						
Q9NP77	SSU72		Yes					
O75915	ARL6IP5		Yes	Yes				
Q3T906	GNPTAB			Yes	Yes			
Q9Y5M8	SRPRB		Yes	Yes				
Q6UW49	SPESP1	Yes						
Q16626	MEA1		Yes					

Q8WWY8	LIPH	Yes					Yes	
Q2UY09	COL28A1	Yes						
Q14108	SCARB2			Yes	Yes	Yes		Yes
Q9HBM1	SPC25		Yes					
Q5SGD2	PPM1L				Yes			
Q9NWK9	ZNHIT6		Yes					
Q5VSG8	MANEAL			Yes				
Q8TCT9	HM13			Yes		Yes		
Q8N108	MIER1		Yes					
Q9ULI3	HEG1	Yes		Yes	Yes	Yes		
Q9H5X1	FAM96A	Yes						
Q8NFU3	TSTD1		Yes					
P11802	CDK4							Yes
Q8N729	NPW	Yes					Yes	
Q92600	RQCD1		Yes					
Q9BZR6	RTN4R	Yes			Yes	Yes		
O15379	HDAC3		Yes					
Q9NQ92	COPRS		Yes					
P22105	TNXB	Yes			Yes			
O95450	ADAMTS2	Yes						
Q29983	MICA	Yes		Yes	Yes	Yes		
Q92667	AKAP1			Yes				

Q49B96	COX19		Yes					
P55212	CASP6							Yes
P15848	ARSB	Yes			Yes			Yes
P33552	CKS2							Yes
P03952	KLKB1	Yes						
B9A064	IGLL5	Yes						
Q9Y5V0	ZNF706		Yes					
Q9Y3B2	EXOSC1		Yes					
Q16718	NDUFA5		Yes					
Q8TDD1	DDX54		Yes					
Q5VU97	CACHD1	Yes		Yes	Yes	Yes		
Q9P0P0	RNF181		Yes					
Q9NRM1	ENAM	Yes						
Q53HL2	CDCA8		Yes					
Q8IXM2	BAP18		Yes					
Q9Y5E6	PCDHB3	Yes		Yes		Yes		
Q16799	RTN1			Yes				
Q15067	ACOX1		Yes					Yes
P15692	VEGFA	Yes					Yes	
P31749	AKT1							Yes
Q9BUW7	C9orf16		Yes					
Q9UHG3	PCYOX1	Yes			Yes			

O76075	DFFB		Yes					
Q92954	PRG4	Yes						
O43760	SYNGR2		Yes	Yes				
Q13724	MOGS			Yes				
P09619	PDGFRB	Yes		Yes	Yes	Yes		
A0AVI2	FER1L5			Yes				
P23470	PTPRG	Yes		Yes	Yes	Yes		
P03951	F11	Yes					Yes	
Q14627	IL13RA2	Yes		Yes	Yes	Yes		
P26374	CHML							Yes
Q9BVK6	TMED9	Yes		Yes	Yes			
P83436	COG7		Yes					
O60575	SPINK4	Yes						
Q6IAA8	LAMTOR1		Yes					
P08962	CD63			Yes	Yes	Yes		Yes
Q9NQX5	NPDC1	Yes		Yes				
Q15726	KISS1	Yes					Yes	
Q9H3H3	C11orf68		Yes					
P35670	ATP7B		Yes	Yes				Yes
O75487	GPC4	Yes			Yes	Yes		
Q9NWY4	C4orf27		Yes					
Q7KYR7	BTN2A1	Yes		Yes	Yes	Yes		

Q7Z7H5	TMED4	Yes		Yes	Yes			
Q9H875	PRKRIP1		Yes					
P13612	ITGA4	Yes		Yes	Yes	Yes		
P29372	MPG		Yes					
P12821	ACE	Yes		Yes	Yes	Yes	Yes	Yes
P01833	PIGR	Yes		Yes		Yes		
Q8NC44	FAM134A			Yes				
Q9H4A6	GOLPH3		Yes					
P04637	TP53		Yes					
Q15375	EPHA7	Yes		Yes		Yes		
Q9H5Y7	SLITRK6	Yes		Yes	Yes	Yes		
P40616	ARL1		Yes					Yes
Q9NRX1	PNO1		Yes					
Q6EEV4	POLR2M		Yes					
P25490	YY1		Yes					
Q96HQ2	CDKN2AIPNL	Yes						
Q9BW71	HIRIP3		Yes					
Q5SRI9	MANEA			Yes				
Q9Y6M7	SLC4A7			Yes	Yes	Yes		
P55083	MFAP4	Yes			Yes			
Q6UWB1	IL27RA	Yes		Yes	Yes	Yes		
Q8NFZ8	CADM4	Yes		Yes	Yes	Yes		

Q7Z5G4	GOLGA7		Yes					
Q96AP7	ESAM	Yes		Yes	Yes	Yes		
Q12893	TMEM115			Yes				
Q9NSI2	FAM207A		Yes					
A0FGR8	ESYT2		Yes	Yes				
O95395	GCNT3			Yes				
Q9UIL1	SCOC		Yes					
Q86WA6	BPHL							Yes
P16871	IL7R	Yes		Yes	Yes	Yes		
Q9H8M7	FAM188A		Yes					
Q16563	SYPL1		Yes	Yes	Yes	Yes		
P57105	SYNJ2BP			Yes				
C9J7I0	UMAD1		Yes					
Q9Y5E7	PCDHB2	Yes		Yes		Yes		
Q9BZP6	CHIA	Yes						
Q5ZPR3	CD276	Yes		Yes	Yes	Yes		
P07902	GALT		Yes					
P28676	GCA		Yes					
Q16773	CCBL1							Yes
Q9UBX1	CTSF	Yes						
Q96HR8	NAF1		Yes					
Q8NFH3	NUP43		Yes					

Q96DE5	ANAPC16		Yes					
P02753	RBP4	Yes					Yes	
O15269	SPTLC1		Yes	Yes				
Q9NY61	AATF		Yes					
Q9H3T3	SEMA6B	Yes		Yes	Yes	Yes		
Q00973	B4GALNT1	Yes		Yes				
Q13277	STX3			Yes				
Q9BXJ1	C1QTNF1	Yes		Yes			Yes	
Q14573	ITPR3			Yes	Yes			
O94910	LPHN1	Yes		Yes		Yes		
Q9HDC9	APMAP		Yes	Yes	Yes			
Q9Y5E9	PCDHB14	Yes		Yes		Yes		
Q9NWS0	PIH1D1		Yes					
Q13586	STIM1	Yes		Yes	Yes	Yes		
Q9HAN9	NMNAT1		Yes					
Q9Y5H1	PCDHGA2	Yes		Yes		Yes		
P25098	ADRBK1							Yes
P13688	CEACAM1	Yes		Yes	Yes	Yes		Yes
P23471	PTPRZ1	Yes		Yes	Yes	Yes		
Q8N6Y2	LRRC17	Yes						
O14817	TSPAN4			Yes	Yes	Yes		
P05408	SCG5	Yes						

O95059	RPP14		Yes					
Q9NRY2	INIP		Yes					
O43653	PSCA					Yes		
Q86U28	ISCA2		Yes					
O76096	CST7	Yes						
Q12884	FAP			Yes	Yes	Yes		
Q8TEM1	NUP210	Yes		Yes	Yes	Yes		
P05412	JUN		Yes					
Q13232	NME3	Yes						
O95274	LYPD3	Yes			Yes	Yes	Yes	
Q8N4P3	HDDC3		Yes					
P20848	SERPINA2	Yes						
P25942	CD40	Yes		Yes	Yes	Yes		Yes
P02686	MBP		Yes					
P00488	F13A1				Yes		Yes	
O75569	PRKRA		Yes					
Q9BUR4	WRAP53		Yes					
Q9Y4P3	TBL2	Yes		Yes				
Q9Y625	GPC6	Yes				Yes		
Q9NPF2	CHST11			Yes	Yes			
Q9UL25	RAB21		Yes					
Q147X3	NAA30		Yes					

P22003	BMP5						Yes	
Q8WYQ3	CHCHD10		Yes					
Q12974	PTP4A2		Yes					
P53801	PTTG1IP	Yes		Yes	Yes	Yes		Yes
Q9NPF0	CD320	Yes		Yes		Yes		
Q13887	KLF5		Yes					Yes
O00534	VWA5A		Yes					
Q14956	GPNMB	Yes		Yes	Yes	Yes		
Q8NHH9	ATL2		Yes	Yes				
P27487	DPP4			Yes	Yes	Yes		
P14927	UQCRB		Yes					
P51693	APLP1	Yes		Yes	Yes	Yes		Yes
Q96KN1	FAM84B		Yes					
O60870	KIN		Yes					
Q969E8	TSR2		Yes					
P52298	NCBP2		Yes					
P61626	LYZ	Yes					Yes	
Q9BYD6	MRPL1		Yes					
Q7Z6R9	TFAP2D		Yes					
Q96KC8	DNAJC1	Yes		Yes				
P05019	IGF1	Yes					Yes	
Q8IXQ3	C9orf40		Yes					

Q9BV19	C1orf50		Yes					
P14406	COX7A2			Yes				Yes
P0DJI9	SAA2	Yes						
Q9UIC8	LCMT1		Yes					
P32320	CDA		Yes					Yes
P09132	SRP19		Yes					
Q12834	CDC20							Yes
Q9H7L9	SUDS3		Yes					
Q99805	TM9SF2	Yes		Yes		Yes		
Q12846	STX4		Yes	Yes				
O43169	CYB5B		Yes	Yes				
P05090	APOD	Yes			Yes		Yes	Yes
Q9BX67	JAM3	Yes		Yes	Yes	Yes		
Q9GZS1	POLR1E		Yes					
Q6UW32	IGFL1	Yes		Yes			Yes	
O75477	ERLIN1			Yes	Yes			
Q16762	TST		Yes					
P20333	TNFRSF1B	Yes		Yes		Yes		
Q9NZ53	PODXL2	Yes		Yes	Yes	Yes	Yes	
Q9NVP2	ASF1B		Yes					
P51946	CCNH							Yes
Q9HD45	TM9SF3	Yes		Yes	Yes	Yes		

Q9P0S2	COX16			Yes				
P62273	RPS29		Yes					
Q8IWT0	ZBTB8OS		Yes					
Q96CX2	KCTD12		Yes					
Q86Y38	XYLT1			Yes				
Q8NB37	PDDC1		Yes					
Q9BRX9	WDR83		Yes					
Q9NWU1	OXSM		Yes					
Q17R31	TATDN3		Yes					
Q5T7V8	GORAB		Yes					
Q15738	NSDHL			Yes				
Q9UFW8	CGGBP1		Yes					
Q03692	COL10A1	Yes						Yes
P62875	POLR2L		Yes					
P48730	CSNK1D		Yes					Yes
Q9Y5H3	PCDHGA10	Yes		Yes		Yes		
P41440	SLC19A1			Yes	Yes	Yes		
Q86TH1	ADAMTSL2	Yes						
Q9NY46	SCN3A			Yes		Yes		
P15018	LIF	Yes					Yes	
Q9Y4W6	AFG3L2			Yes				
Q9HD47	RANGRF		Yes					

Q9UKZ4	TENM1			Yes	Yes	Yes		
Q9Y639	NPTN	Yes		Yes	Yes	Yes		
Q75N90	FBN3	Yes						
Q9BVM2	DPCD		Yes					
Q9H461	FZD8	Yes		Yes		Yes		
Q6UWI4	SHISA2	Yes		Yes				
O15551	CLDN3			Yes		Yes		Yes
P78552	IL13RA1	Yes		Yes	Yes	Yes		
O14493	CLDN4			Yes		Yes		Yes
P21860	ERBB3	Yes		Yes	Yes	Yes		
P43307	SSR1	Yes		Yes	Yes	Yes		
P06731	CEACAM5	Yes				Yes		Yes
Q5GFL6	VWA2	Yes						
Q8N434	SVOPL		Yes	Yes		Yes		
A6NHX0	GATSL2		Yes					
Q9HCL0	PCDH18	Yes		Yes	Yes	Yes		
Q96IV0	NGLY1		Yes					
O14646	CHD1							Yes
Q8WTV0	SCARB1			Yes	Yes	Yes		Yes
Q12955	ANK3							Yes
Q9NY15	STAB1	Yes		Yes	Yes	Yes		
Q8WXG9	GPR98			Yes		Yes		

O43716	GATC		Yes					
Q07654	TFF3	Yes						
P04196	HRG	Yes					Yes	
P21583	KITLG	Yes		Yes	Yes	Yes	Yes	
Q9BSR8	YIPF4			Yes				
Q8N5P1	ZC3H8		Yes					
P14621	ACYP2		Yes					Yes
Q96I23	PYURF		Yes					
P11274	BCR							Yes
Q8IV50	LYSMD2		Yes					
Q9BUL9	RPP25		Yes					
Q9C004	SPRY4		Yes					
Q9NRR1	CYTL1	Yes						
Q9C002	NMES1			Yes				
Q5VW36	FOCAD		Yes					
Q92791	LEPREL4	Yes			Yes			
P00450	CP	Yes			Yes		Yes	Yes
Q9Y679	AUP1			Yes				Yes
Q9Y3C0	CCDC53		Yes					
Q96IJ6	GMPPA		Yes					
O43567	RNF13			Yes	Yes	Yes		
Q06828	FMOD	Yes						

Q14031	COL4A6	Yes					Yes	Yes
Q9H7P6	MVB12B		Yes					
Q7Z4G1	COMMD6		Yes					
Q96C57	C12orf43		Yes					
O15391	YY2		Yes					
Q9P0P8	C6orf203		Yes					
O14498	ISLR	Yes			Yes			
P0DP57		Yes						
Q9UNK0	STX8			Yes				
Q13361	MFAP5	Yes			Yes		Yes	
Q9Y2B1	TMEM5			Yes				
Q8TDP1	RNASEH2C		Yes					
Q96A73	KIAA1191		Yes					
P46531	NOTCH1	Yes			Yes	Yes		
Q8NFP9	NBEA		Yes					
Q9UEU0	VTI1B			Yes				
Q96BM9	ARL8A		Yes					
Q8IWA5	SLC44A2		Yes	Yes	Yes	Yes		
Q9UHF1	EGFL7	Yes						
Q00887	PSG9	Yes						
Q13433	SLC39A6	Yes		Yes	Yes	Yes		
Q92686	NRGN		Yes					

O15173	PGRMC2			Yes				
O76080	ZFAND5		Yes					
P16234	PDGFRA	Yes		Yes	Yes	Yes		
Q8N5K1	CISD2			Yes				
Q9H4A5	GOLPH3L		Yes					
Q9UL33	TRAPPC2L		Yes					
Q8WW01	TSEN15		Yes					
Q9UKF2	ADAM30	Yes		Yes		Yes		
Q9NVM6	DNAJC17		Yes					
P47929	LGALS7		Yes					
Q9Y2Y0	ARL2BP		Yes					
Q9BTY7	HGH1		Yes					
P32241	VIPR1	Yes		Yes	Yes	Yes		
Q13542	EIF4EBP2		Yes					
Q14435	GALNT3			Yes				
Q13278	RIG		Yes					
P09681	GIP	Yes					Yes	
O76024	WFS1			Yes				
Q9NQX7	ITM2C		Yes	Yes		Yes		
Q9BYC5	FUT8			Yes				
O95445	APOM	Yes						
P36542	ATP5C1							Yes

Q8NEW0	SLC30A7			Yes				
Q92824	PCSK5	Yes		Yes		Yes		
Q8IXL7	MSRB3	Yes						
Q14244	MAP7		Yes					
Q99102	MUC4	Yes		Yes	Yes	Yes		
Q96PC5	MIA2	Yes		Yes				
Q8NC56	LEMD2			Yes	Yes			
Q96SZ5	ADO		Yes					
Q9UIV8	SERPINB13			Yes				
P43007	SLC1A4		Yes	Yes	Yes	Yes		
O95352	ATG7		Yes					
Q96I82	KAZALD1	Yes						
Q9NX70	MED29		Yes					
Q6J9G0	STYK1			Yes				
C9JLW8	FAM195B		Yes					
Q9BUH6	C9orf142		Yes					
Q9Y2G5	POFUT2	Yes			Yes			
P0C7U0	ELFN1	Yes		Yes	Yes	Yes		
P00403	MT-CO2			Yes				
Q6I9Y2	THOC7		Yes					
A6NDU8	C5orf51		Yes					
Q7Z7G0	ABI3BP	Yes			Yes			

Q9H1K1	ISCU		Yes					
P49184	DNASE1L1	Yes			Yes			
Q68CQ7	GLT8D1			Yes	Yes			
P53384	NUBP1		Yes					
P28827	PTPRM	Yes		Yes	Yes	Yes		
Q6IA86	ELP2		Yes					
Q15043	SLC39A14	Yes		Yes	Yes	Yes		
Q9UJZ1	STOML2		Yes					
O43657	TSPAN6			Yes	Yes	Yes		
Q9UJX2	CDC23		Yes					
Q8N8R5	C2orf69	Yes						
Q9H1A4	ANAPC1		Yes					
P51793	CLCN4		Yes	Yes				Yes
P34741	SDC2	Yes		Yes		Yes		
Q7Z7K0	CMC1		Yes					
Q9HC56	PCDH9	Yes		Yes	Yes	Yes		
Q9Y421	FAM32A		Yes					
P16471	PRLR	Yes		Yes		Yes		
O00488	ZNF593		Yes					
Q15773	MLF2		Yes					
P53803	POLR2K		Yes					
Q96MH2	HEXIM2		Yes					

Q6ZXV5	TMTC3			Yes				
O15182	CETN3		Yes					Yes
Q5T601	GPR110	Yes		Yes	Yes	Yes		
Q13635	PTCH1			Yes	Yes	Yes		
O15118	NPC1	Yes		Yes	Yes	Yes		
Q9NV35	NUDT15		Yes					
O43184	ADAM12	Yes		Yes	Yes	Yes	Yes	
Q6P161	MRPL54		Yes					
Q8IZK6	MCOLN2			Yes				
Q8WZ82	OVCA2		Yes					
Q2VWP7	PRTG	Yes		Yes		Yes		
Q93033	CD101	Yes		Yes	Yes	Yes		
P53701	HCCS		Yes					
Q02083	NAAA	Yes		Yes	Yes			
O95295	SNAPIN		Yes					
P09038	FGF2						Yes	
Q9UN73	PCDHA6	Yes			Yes	Yes		
Q13535	ATR							Yes
Q13015	MLLT11		Yes					
Q8NBI6	XXYLT1			Yes				
Q11201	ST3GAL1			Yes	Yes			
Q9ULH0	KIDINS220			Yes	Yes			

Q9NWQ9	C14orf119		Yes					
Q9Y3I1	FBXO7		Yes					
Q5T440	IBA57		Yes					
P14209	CD99	Yes		Yes				
P06727	APOA4	Yes						Yes
Q9NW97	TMEM51			Yes				
O75508	CLDN11			Yes		Yes		
Q96NU0	CNTNAP3B	Yes		Yes		Yes		
O95167	NDUFA3			Yes				
P16473	TSHR	Yes		Yes	Yes	Yes		
P05305	EDN1	Yes					Yes	
O15533	TAPBP	Yes		Yes				
Q8WUB8	PHF10		Yes					
Q04941	PLP2		Yes	Yes	Yes			
O14662	STX16			Yes				
Q9P121	NTM	Yes			Yes	Yes		
P39059	COL15A1	Yes						Yes
Q9H160	ING2		Yes					
P37287	PIGA			Yes				
Q9UBN6	TNFRSF10D	Yes		Yes		Yes		
Q9Y6M5	SLC30A1			Yes		Yes		
P35612	ADD2							Yes

Q2M3G0	ABCB5			Yes		Yes		
Q9Y605	MRFAP1		Yes					
Q9NZS9	BFAR		Yes	Yes				
Q6ZNA5	FRRS1	Yes		Yes		Yes		
Q9UJM3	ERRFI1		Yes					
Q8N5M4	TTC9C		Yes					
Q9NYQ8	FAT2	Yes		Yes		Yes		
O60635	TSPAN1			Yes	Yes	Yes		
Q96CS2	HAUS1		Yes					
Q9NNW5	WDR6		Yes					
O95631	NTN1	Yes					Yes	
Q9NXS2	QPCTL		Yes	Yes				
P23025	XPA		Yes					
P17342	NPR3	Yes		Yes	Yes	Yes		
O95750	FGF19	Yes		Yes			Yes	
Q8WWZ7	ABCA5		Yes	Yes	Yes	Yes		
Q03188	CENPC							Yes
P11310	ACADM		Yes					Yes
Q32ZL2	LPPR5			Yes		Yes		
P50440	GATM		Yes					
Q92729	PTPRU	Yes		Yes		Yes		
Q6PJG6	BRAT1		Yes					

P51571	SSR4	Yes						
Q96EX1	SMIM12			Yes				
Q9BXT2	CACNG6			Yes	Yes	Yes		
Q9HCU8	POLD4		Yes					
O95490	LPHN2	Yes		Yes	Yes	Yes		
Q9H1J7	WNT5B	Yes			Yes			
P15502	ELN	Yes		Yes				
Q15012	LAPTM4A			Yes				
Q8WXF8	DEDD2		Yes					
Q15041	ARL6IP1			Yes				
O14757	CHEK1							Yes
Q96PI1	SPRR4		Yes					
O60831	PRAF2			Yes				
Q9BTT4	MED10		Yes					
Q6UXH8	CCBE1	Yes						
Q96HR9	REEP6			Yes				
Q86VR2	FAM134C			Yes				
Q9BZM4	ULBP3	Yes			Yes	Yes		
P00797	REN	Yes					Yes	
Q8NBN3	TMEM87A	Yes		Yes	Yes	Yes		
Q8WXD2	SCG3	Yes						
Q8IV56	PRR15		Yes					

Q8IWU2	LMTK2	Yes		Yes				
O15321	TM9SF1	Yes		Yes	Yes	Yes		
Q9Y6G3	MRPL42		Yes					
Q96PE7	MCEE		Yes					
P15954	COX7C		Yes					Yes
Q96MM7	HS6ST2			Yes				
Q8NFT8	DNER	Yes		Yes	Yes	Yes		
Q6X4U4	SOSTDC1	Yes						
O95478	NSA2		Yes					
P04155	TFF1	Yes						
Q8NFW1	COL22A1	Yes						
Q96SY0	VWA9		Yes					
P18075	BMP7	Yes					Yes	Yes
P19801	AOC1	Yes						Yes
Q8NEF3	CCDC112		Yes					
Q96S97	MYADM		Yes	Yes		Yes		
Q9BYV8	CEP41		Yes					
Q5T8D3	ACBD5		Yes	Yes				
Q9ULX9	MAFF		Yes					
O95551	TDP2		Yes					
O95498	VNN2	Yes		Yes	Yes	Yes		
Q01973	ROR1	Yes		Yes	Yes	Yes		

Q9Y2Q9	MRPS28		Yes					
O60243	HS6ST1			Yes	Yes			
Q8IW45	CARKD	Yes		Yes				
Q8NBN7	RDH13	Yes						
O43155	FLRT2	Yes		Yes	Yes	Yes		
Q9BUA3	C11orf84		Yes					
Q96L58	B3GALT6			Yes				
Q9BRQ3	NUDT22		Yes					
Q53RD9	FBLN7	Yes						
Q16854	DGUOK		Yes					
Q96CS3	FAF2		Yes					
P16157	ANK1							Yes
Q8WXD5	GEMIN6		Yes					
Q9UBT3	DKK4	Yes						
Q9NZH0	GPRC5B	Yes		Yes		Yes		
O00399	DCTN6		Yes					
Q5VZ72	IZUMO3	Yes		Yes		Yes		
Q14318	FKBP8			Yes				
Q9UPZ6	THSD7A	Yes		Yes	Yes	Yes		
Q6UWY0	ARSK	Yes		Yes				
Q99732	LITAF		Yes					
P23297	S100A1		Yes					

Q5JWF8	ACTL10	Yes						
Q96DR5	BPIFA2	Yes						
Q9UQB3	CTNND2							Yes
P51790	CLCN3		Yes	Yes				Yes
Q68BL8	OLFML2B	Yes		Yes	Yes			
Q96AZ6	ISG20		Yes					
O60637	TSPAN3			Yes	Yes	Yes		
Q9H467	CUEDC2		Yes					
Q969J3	LOH12CR1		Yes					
P42773	CDKN2C							Yes
P62068	USP46		Yes					
O15354	GPR37	Yes		Yes		Yes		
Q8TDX5	ACMSD			Yes				
O95639	CPSF4		Yes					
O43920	NDUFS5		Yes					
Q96GM8	TOE1		Yes					
Q9NPL8	TIMMDC1			Yes				
Q969X1	TMBIM1		Yes	Yes				
Q99836	MYD88		Yes					
P61952	GNG11		Yes					
O15127	SCAMP2			Yes				
P21709	EPHA1	Yes		Yes	Yes	Yes		

O43736	ITM2A		Yes	Yes				
P37173	TGFBR2	Yes		Yes	Yes	Yes		
Q04912	MST1R	Yes		Yes	Yes	Yes		
P18850	ATF6		Yes					
Q01523	DEFA5	Yes						
Q9Y274	ST3GAL6			Yes	Yes			
P58511	SMIM11			Yes				
P33261	CYP2C19	Yes						
Q9NRX5	SERINC1			Yes	Yes	Yes		
Q9Y6X5	ENPP4	Yes		Yes	Yes	Yes		
P15336	ATF2		Yes					Yes
Q02383	SEMG2	Yes						
Q9H4G4	GLIPR2		Yes					
Q9BYD1	MRPL13		Yes					
P78556	CCL20	Yes					Yes	
Q8TE58	ADAMTS15	Yes			Yes			
Q15363	TMED2	Yes		Yes				
Q96HD1	CRELD1	Yes		Yes				
Q9NQS1	AVEN		Yes					
Q9UBP0	SPAST		Yes	Yes				
P17405	SMPD1		Yes	Yes	Yes			
Q7Z309	FAM122B		Yes					

Q86Z23	C1QL4	Yes						
Q6P5R6	RPL22L1		Yes					
Q2VYF4	LETM2		Yes	Yes				
P08F94	PKHD1			Yes		Yes		
P51648	ALDH3A2			Yes				Yes
O94901	SUN1			Yes	Yes			
Q9BYI3	FAM126A		Yes					
Q9BRR6	ADPGK	Yes						
P0C7P4	UQCRFS1P1	Yes						
O00555	CACNA1A			Yes				Yes
ENST00000628037_NCI-H23_Mis:R33Q	PTGER3			Yes	Yes	Yes		
P50053	KHK		Yes					
P02775	PPBP	Yes					Yes	
O95229	ZWINT		Yes					
Q92581	SLC9A6	Yes		Yes		Yes		
Q9UJH8	METRN	Yes						
Q9P2N4	ADAMTS9	Yes						
O95528	SLC2A10			Yes		Yes		
Q12866	MERTK	Yes		Yes	Yes	Yes		
Q6V0I7	FAT4	Yes			Yes	Yes	Yes	
P41134	ID1		Yes					
Q96EZ8	MCRS1		Yes					

P38398	BRCA1							Yes
O75899	GABBR2	Yes		Yes		Yes		
P17676	CEBPB		Yes					Yes
Q9NP58	ABCB6			Yes				
P13671	C6	Yes						Yes
Q9H7E2	TDRD3		Yes					
Q15126	PMVK		Yes					
Q9HA38	ZMAT3		Yes					
P79522	PRR3		Yes					
P38570	ITGAE	Yes		Yes	Yes	Yes		
Q92793	CREBBP							Yes
Q9UKX5	ITGA11	Yes		Yes	Yes	Yes		
Q8N9I0	SYT2			Yes				
Q9BXJ0	C1QTNF5	Yes					Yes	
Q9BZL1	UBL5		Yes					
Q16790	CA9	Yes		Yes				Yes
P82933	MRPS9		Yes					
Q9Y2R9	MRPS7		Yes					
Q99717	SMAD5		Yes					
Q9Y5F8	PCDHGB7	Yes		Yes	Yes	Yes		
Q13342	SP140		Yes					
Q86YD3	TMEM25	Yes		Yes	Yes	Yes		

O75711	SCRG1	Yes		Yes				
P0DPB6			Yes					
Q8NHG8	ZNRF2		Yes					
Q99933	BAG1							Yes
Q96QV1	HHIP	Yes			Yes			
A8K0S8	MEIS3P2		Yes					
Q9Y5T4	DNAJC15		Yes	Yes				
Q86WC4	OSTM1	Yes		Yes	Yes	Yes		
Q9UKU6	TRHDE			Yes	Yes	Yes		
Q5SZJ8	BEND6		Yes					
Q5SZK8	FREM2	Yes		Yes	Yes	Yes		
Q9NX65	ZSCAN32		Yes					
Q9NWZ8	GEMIN8		Yes					
Q9HBX9	RXFP1	Yes		Yes		Yes		
P56277	CMC4		Yes					
Q9H1Z9	TSPAN10			Yes				
Q9HAR2	LPHN3	Yes		Yes		Yes		
P49675	STAR		Yes					
Q9UM47	NOTCH3	Yes				Yes		
Q9BYN8	MRPS26		Yes					
Q8N129	CNPY4	Yes						
Q9Y6V7	DDX49		Yes					

P04921	GYPC		Yes	Yes		Yes		
O60566	BUB1B							Yes
O15484	CAPN5		Yes					Yes
Q6UXM1	LRIG3	Yes		Yes		Yes		
Q9Y394	DHRS7			Yes				
Q8N6G6	ADAMTSL1	Yes						
Q14691	GINS1		Yes					
Q9UHJ6	SHPK		Yes					
O14656	TOR1A	Yes						
O60242	BAI3	Yes		Yes		Yes		Yes
Q9BZC7	ABCA2			Yes	Yes	Yes		Yes
Q969M3	YIPF5		Yes	Yes				
Q5SR56	HIATL1			Yes				
Q9NPC2	KCNK9	Yes		Yes				
A1KXE4	FAM168B		Yes					
Q9P2X0	DPM3	Yes		Yes				
P05106	ITGB3	Yes		Yes	Yes	Yes		
Q9H4D0	CLSTN2	Yes		Yes		Yes		
Q8WZA1	POMGNT1		Yes					
Q9Y694	SLC22A7		Yes	Yes		Yes		
Q96DD7	SHISA4	Yes		Yes		Yes		
Q9UBS3	DNAJB9	Yes						

Q9NZE8	MRPL35		Yes					
Q96N03	VSTM2L	Yes						
P35548	MSX2		Yes					
Q9GZV7	HAPLN2	Yes						
P28907	CD38			Yes	Yes	Yes		Yes
P20783	NTF3	Yes					Yes	
Q504U0	C4orf46		Yes					
P32926	DSG3	Yes				Yes		
P48551	IFNAR2			Yes	Yes	Yes		
Q96DA6	DNAJC19			Yes				
Q9NQS3	PVRL3	Yes		Yes	Yes	Yes		
P55789	GFER		Yes					
Q9BSY4	CHCHD5		Yes					
Q96JJ7	TMX3	Yes		Yes	Yes	Yes		
O60487	MPZL2	Yes		Yes	Yes	Yes		
Q8N4M1	SLC44A3		Yes	Yes		Yes		
Q9Y3A6	TMED5	Yes		Yes				
Q7LFX5	CHST15		Yes	Yes				
Q9C0B7	TANGO6		Yes					
Q9BVC4	MLST8		Yes					
P35475	IDUA	Yes						
O95497	VNN1	Yes			Yes	Yes		

Q9HCJ1	ANKH			Yes		Yes		
O95396	MOCS3		Yes					
Q96GE4	CEP95		Yes					
Q96DX5	ASB9		Yes					
Q9UMX3	BOK		Yes					Yes
Q9H0X4	ITFG3		Yes	Yes	Yes			
Q8IWB9	TEX2			Yes				
Q9Y6Q6	TNFRSF11A	Yes		Yes	Yes	Yes		
Q16647	PTGIS	Yes		Yes				
O75027	ABCB7			Yes				Yes
Q99623	PHB2		Yes		Yes			
Q05586	GRIN1	Yes		Yes		Yes		
O95897	OLFM2	Yes					Yes	
Q96N06	SPATA33		Yes					
Q5T742	C10orf25	Yes						
O75446	SAP30		Yes					
Q15165	PON2	Yes			Yes		Yes	
Q13796	SHROOM2							Yes
Q16853	AOC3			Yes	Yes	Yes		
O95980	RECK	Yes		Yes	Yes	Yes		
Q92567	FAM168A		Yes					
P51636	CAV2		Yes	Yes				Yes

Q9NU53	GINM1	Yes		Yes		Yes		
Q9UIB8	CD84	Yes		Yes	Yes	Yes		
O15496	PLA2G10	Yes		Yes			Yes	
Q9NVA4	TMEM184C			Yes				
Q4V9L6	TMEM119	Yes		Yes				
P54922	ADPRH							Yes
Q9BQE5	APOL2		Yes	Yes				
Q9BXR5	TLR10	Yes		Yes		Yes		
P35247	SFTPD	Yes					Yes	
Q9Y2R0	COA3			Yes				
Q9Y2P8	RCL1		Yes					
P27930	IL1R2	Yes		Yes	Yes	Yes		
Q02447	SP3		Yes					
O95096	NKX2-2		Yes					
Q5JS37	NHLRC3	Yes		Yes				
Q9NY35	CLDND1			Yes	Yes	Yes		
Q9P0L9	PKD2L1			Yes		Yes		
O60279	SUSD5	Yes		Yes	Yes	Yes		
Q14162	SCARF1	Yes				Yes		
Q92851	CASP10							Yes
Q13190	STX5			Yes				
Q9HAB3	SLC52A2			Yes		Yes		

Q12767	KIAA0195		Yes	Yes	Yes			
O60840	CACNA1F			Yes				Yes
O43819	SCO2		Yes					
Q9NRN9	METTL5		Yes					
Q96BX8	MOB3A		Yes					
Q86TN4	TRPT1		Yes					
P22749	GNLY	Yes						
Q99633	PRPF18		Yes					
Q8N8A6	DDX51		Yes					
Q13007	IL24	Yes					Yes	
Q9BRA0	NAA38		Yes					
Q9UP95	SLC12A4			Yes		Yes		
P51170	SCNN1G			Yes		Yes		
O43157	PLXNB1	Yes			Yes	Yes		
Q9Y320	TMX2			Yes				
Q9Y644	RFNG	Yes		Yes	Yes			
Q0P641	C2orf80		Yes					
Q9BYG0	B3GNT5			Yes				
O43294	TGFB1I1		Yes					
O00483	NDUFA4			Yes				
ENST00000613718_DAN-G_Mis:F127L	CSH1		Yes				Yes	
Q9HBU6	ETNK1		Yes	Yes				

O95390	GDF11	Yes					Yes	
Q2VPA4	CR1L	Yes						
Q9H1Z4	WDR13		Yes		Yes			
A0A075B6H9	IGLV4-69	Yes						
ENST00000391814_CAPAN-1_Mis:D371H	SHANK1						Yes	
Q9BW61	DDA1		Yes					
P43353	ALDH3B1		Yes					Yes
Q07092	COL16A1	Yes						Yes
Q8NBL1	POGLUT1	Yes						
Q9BQE4	VIMP			Yes				
Q96AM1	MRGPRF		Yes	Yes	Yes	Yes		
Q96JA1	LRIG1	Yes		Yes		Yes		
P18564	ITGB6	Yes		Yes	Yes	Yes		
Q8N300	CCDC23		Yes					
Q9Y5Z9	UBIAD1		Yes	Yes				
O15427	SLC16A3			Yes				
Q6PJF5	RHBDF2			Yes	Yes	Yes		
P53539	FOSB		Yes					
Q96A00	PPP1R14A		Yes					
ENST00000373957_SW620_Mis:S1942N	PCDH15	Yes		Yes				
Q9NXV2	KCTD5		Yes					

P06870	KLK1	Yes						
P0DPB5			Yes					
Q9NWQ8	PAG1			Yes				
Q8TAA1	RNASE11	Yes						
Q8WUH6	TMEM263			Yes				
Q9UBB6	NCDN		Yes					
P50461	CSRP3		Yes					
P98173	FAM3A			Yes				
Q13901	C1D		Yes					
O75054	IGSF3	Yes		Yes	Yes	Yes		
Q9NRB3	CHST12			Yes	Yes			
Q6ZTI6	FAM101A		Yes					
P12319	FCER1A	Yes		Yes		Yes		
Q92544	TM9SF4	Yes		Yes		Yes		
O14495	PPAP2B			Yes	Yes	Yes		
Q14050	COL9A3	Yes					Yes	Yes
P52797	EFNA3	Yes				Yes	Yes	
P54289	CACNA2D1	Yes			Yes			Yes
P23560	BDNF	Yes			Yes		Yes	Yes
Q16698	DECR1		Yes					
P60022	DEFB1	Yes					Yes	
Q9BQ51	PDCD1LG2	Yes		Yes	Yes	Yes		

Q06418	TYRO3	Yes		Yes	Yes	Yes		
Q9NXW2	DNAJB12		Yes	Yes				
Q7Z7E8	UBE2Q1		Yes					
Q8N428	GALNT16			Yes				
Q9Y3E5	PTRH2			Yes				
Q13017	ARHGAP5							Yes
Q8NBP0	TTC13	Yes		Yes				
Q9UHR6	ZNHIT2							Yes
Q9Y6T7	DGKB							Yes
Q96S16	JMJD8	Yes						
Q96A83	COL26A1	Yes		Yes	Yes			
Q9NXS3	KLHL28		Yes					
P36222	CHI3L1	Yes						Yes
Q8NA58	PNLDC1	Yes						
O95182	NDUFA7		Yes					
Q8TCZ2	CD99L2	Yes		Yes				
Q9H172	ABCG4			Yes		Yes		
Q13489	BIRC3							Yes
Q9H000	MKRN2		Yes					
Q10589	BST2			Yes	Yes	Yes		Yes
Q7Z5N4	SDK1			Yes	Yes	Yes		
Q8IY95	TMEM192			Yes				

P51828	ADCY7			Yes	Yes	Yes		Yes
P13686	ACP5	Yes						Yes
Q9UHY8	FEZ2		Yes					
Q12860	CNTN1	Yes			Yes	Yes		Yes
Q9H6E4	CCDC134	Yes						
Q8N6G5	CSGALNACT2			Yes				
O60613	SEPTIN5	Yes						
P36268	GGT2			Yes				
Q9ULZ1	APLN	Yes		Yes			Yes	
Q8N8Q8	COX18		Yes	Yes				
P00746	CFD	Yes						
Q9H159	CDH19	Yes		Yes	Yes	Yes		
P04278	SHBG	Yes					Yes	
P78540	ARG2		Yes					Yes
Q9BVW5	TIPIN		Yes					
Q6NW29	RWDD4		Yes					
P21439	ABCB4		Yes	Yes		Yes		
ENST00000409700_NCI-H23_Mis:D742Y	PCDHAC1	Yes		Yes		Yes		
Q99808	SLC29A1			Yes	Yes	Yes		
P14151	SELL			Yes	Yes	Yes		
Q9H8M9	EVA1A		Yes	Yes				
O94817	ATG12		Yes					

P17706	PTPN2		Yes	Yes				
O94966	USP19			Yes				
P78412	IRX6		Yes					
Q9P273	TENM3			Yes	Yes	Yes		
Q8NGR9	OR1N2		Yes	Yes		Yes		
Q96IQ7	VSIG2	Yes		Yes		Yes		
O75694	NUP155		Yes					
A0A0A6YYD4	TRBV13	Yes		Yes				
O94919	ENDOD1	Yes		Yes				
Q9UHQ9	CYB5R1			Yes				
Q8WWZ3	EDARADD		Yes					
P10600	TGFB3	Yes					Yes	
Q8IVB4	SLC9A9		Yes	Yes	Yes			
P27701	CD82			Yes	Yes	Yes		
Q9H013	ADAM19			Yes	Yes	Yes		
Q9Y2B9	PKIG		Yes					
O15072	ADAMTS3	Yes						
Q9NRZ5	AGPAT4			Yes				
O43708	GSTZ1		Yes					
Q13873	BMPR2	Yes		Yes	Yes	Yes		Yes
Q02742	GCNT1			Yes				
Q9NZU1	FLRT1	Yes		Yes	Yes	Yes		

P14780	MMP9	Yes					Yes	
Q96J65	ABCC12		Yes	Yes		Yes		
O14896	IRF6		Yes					
Q6RW13	AGTRAP			Yes				
Q9Y2C3	B3GALT5			Yes				
P07204	THBD	Yes		Yes	Yes	Yes		
A1L4H1	SSC5D	Yes						
Q9NYU1	UGGT2	Yes			Yes			
Q9HCK4	ROBO2	Yes		Yes		Yes		
O43182	ARHGAP6							Yes
P0DMQ5	INAFM2		Yes	Yes				
Q99608	NDN		Yes					
Q96EG3	ZNF837		Yes					
P36896	ACVR1B	Yes		Yes		Yes		Yes
Q8WUT9	SLC25A43			Yes				
O60499	STX10			Yes				
P26012	ITGB8	Yes		Yes	Yes	Yes		
Q14249	ENDOG		Yes					
Q9NS69	TOMM22			Yes				
P49427	CDC34							Yes
Q9NPA0	EMC7	Yes		Yes				
Q9NQ84	GPRC5C	Yes		Yes		Yes		

P98194	ATP2C1			Yes				
Q04771	ACVR1	Yes		Yes	Yes	Yes		Yes
Q5SY80	C1orf101	Yes		Yes		Yes		
O60927	PPP1R11		Yes					
Q8N5G0	SMIM20			Yes				
Q8IUL8	CILP2	Yes						
Q14145	KEAP1		Yes					
P43155	CRAT							Yes
P49914	MTHFS		Yes					
Q9H628	RERGL		Yes					
Q8IWD4	CCDC117		Yes					
P14415	ATP1B2		Yes	Yes	Yes	Yes		Yes
B2RXF0	TMEM229A		Yes	Yes				
Q9UER7	DAXX							Yes
O60762	DPM1		Yes					
P15090	FABP4		Yes					
Q5VTU8	ATP5EP2		Yes					
Q16890	TPD52L1		Yes					
Q6V0L0	CYP26C1			Yes				
P53816	PLA2G16			Yes				
Q6P9A2	GALNT18			Yes				
P40200	CD96			Yes	Yes	Yes		

P78325	ADAM8	Yes		Yes	Yes	Yes		Yes
O94759	TRPM2			Yes				
Q4ZHG4	FNDC1	Yes						
Q8TAA9	VANGL1			Yes				
O60741	HCN1			Yes				
P0CE67	C3orf79		Yes					
Q9UIG8	SLCO3A1			Yes	Yes	Yes		
Q9C0C6	CIPC		Yes					
Q9NYQ6	CELSR1	Yes		Yes	Yes	Yes		
Q8N7U9	LINC00469		Yes					
Q5VYY2	LIPM	Yes						
P09919	CSF3	Yes					Yes	Yes
Q6IBW4	NCAPH2		Yes					
P48061	CXCL12	Yes					Yes	
Q9UJ14	GGT7		Yes	Yes	Yes	Yes		
A0A1B0GVG4				Yes				
Q6PJG9	LRFN4	Yes		Yes		Yes		
Q9NXG6	P4HTM		Yes	Yes				
Q6NSI3	FAM53A		Yes					
Q5XXA6	ANO1		Yes	Yes		Yes		
Q9BYB4	GNB1L		Yes					
P05496	ATP5G1			Yes				Yes

Q86SQ3	EMR4P	Yes		Yes		Yes		
Q9H147	DNTTIP1		Yes					
Q7Z449	CYP2U1		Yes	Yes				
Q9HCN8	SDF2L1	Yes		Yes				
P12829	MYL4		Yes					
P33527	ABCC1		Yes	Yes	Yes	Yes		
Q92519	TRIB2		Yes					
Q6ZP68	ATP11AUN		Yes					
Q52LA3	LIN52		Yes					
Q8TCF1	ZFAND1		Yes					
Q9H9B4	SFXN1			Yes				
Q08828	ADCY1		Yes	Yes				Yes
Q12951	FOXI1		Yes					
P10645	CHGA	Yes			Yes			Yes
Q9BY67	CADM1	Yes		Yes	Yes	Yes		
Q5T4F7	SFRP5	Yes						
P05154	SERPINA5	Yes						
Q9NXX6	NSMCE4A		Yes					
Q99470	SDF2	Yes						
P20226	TBP		Yes					
Q7Z7M0	MEGF8	Yes		Yes	Yes	Yes		
Q96B49	TOMM6		Yes					

Q53FT3	C11orf73		Yes					
Q08AG7	MZT1		Yes					
P35790	CHKA							Yes
P30990	NTS	Yes					Yes	
Q9HC73	CRLF2	Yes		Yes	Yes	Yes		
Q9NZD8	SPG21		Yes					
Q9BXJ4	C1QTNF3	Yes						
Q63HM2	PCNXL4		Yes	Yes				
Q1L6U9	MSMP	Yes		Yes				
Q96MU8	KREMEN1	Yes		Yes		Yes		
P31151	S100A7		Yes					
Q8N0T1	C8orf59		Yes					
O43609	SPRY1		Yes					
O75954	TSPAN9			Yes	Yes	Yes		
Q08334	IL10RB	Yes		Yes	Yes	Yes		

Table S3 Significant differentially expressed secreted proteins between cancer-associated fibroblasts and cancer cell lines

T-test highlighted the significant differentially expressed secreted proteins and multiple testing was corrected for with Storey's q value.

**Accession**	**Gene**	**Log2(CAF/Cancer)**	**pvalue**	**BH**	**Q**
P34741	SDC2	4.911298288	0.016395	0.088434	0.039066
Q14573	ITPR3	4.357045568	0.020057	0.097373	0.043014
Q2UY09	COL28A1	4.208882649	1.38E-06	0.000509	0.000225
ENST00000472056_NCI-H23_Mis:T117M	COL6A3	3.62150803	0.019676	0.096616	0.04268
Q9H1J7	WNT5B	3.583940469	0.008388	0.056053	0.024761
P02461	COL3A1	3.432878112	0.013316	0.077515	0.034242
Q12841	FSTL1	3.383233853	4.13E-09	1.06E-05	4.70E-06
P02452	COL1A1	3.360243445	0.003593	0.03325	0.014688
Q9HC97	GPR35	3.272064472	0.018628	0.094074	0.041557
P08123	COL1A2	3.032622874	0.014159	0.080424	0.035527
Q8N130	SLC34A3	3.013666984	0.001193	0.018604	0.008218
P35442	THBS2	2.980063898	0.007234	0.050714	0.022403
Q16270	IGFBP7	2.938129712	0.009069	0.059074	0.026096
Q9NRN5	OLFML3	2.913966041	0.01133	0.069082	0.030517
P35555	FBN1	2.912728866	0.000254	0.008487	0.003749
Q6UXH9	PAMR1	2.776993909	0.007908	0.05383	0.023779

P12110	COL6A2	2.74101887	0.020484	0.09776	0.043185
Q9UBP4	DKK3	2.737543735	0.019938	0.097373	0.043014
P35247	SFTPD	2.705605848	0.005239	0.040851	0.018046
Q969M3	YIPF5	2.684678677	0.001301	0.019131	0.008451
P09486	SPARC	2.625824951	0.009213	0.059713	0.026378
Q13635	PTCH1	2.573554194	0.001525	0.020984	0.00927
P05997	COL5A2	2.507409241	0.000369	0.009989	0.004413
P02458	COL2A1	2.492346831	0.010006	0.062949	0.027808
P21246	PTN	2.420509583	0.020517	0.09776	0.043185
Q96D15	RCN3	2.378156015	0.015092	0.082883	0.036614
P14210	HGF	2.355900372	0.021609	0.100031	0.044189
Q9Y6C2	EMILIN1	2.282746405	0.002251	0.025717	0.01136
Q5JWF8	ACTL10	2.266085307	0.012702	0.07513	0.033189
P20908	COL5A1	2.243177351	0.014638	0.08152	0.036012
P09493	TPM1	2.240017086	0.004137	0.036175	0.01598
O14495	PPAP2B	2.127458758	0.000444	0.01032	0.004559
P12109	COL6A1	2.09126068	0.003479	0.032668	0.014431
P27658	COL8A1	2.080279168	0.004364	0.037198	0.016432
O95967	EFEMP2	2.0536678	0.002723	0.028093	0.01241
Q14766	LTBP1	2.029546331	0.022099	0.101566	0.044867
Q9Y646	CPQ	1.971848938	0.007056	0.050151	0.022154
O43852	CALU	1.963013509	3.99E-07	0.000241	0.000107

Q07954	LRP1	1.962795216	0.003058	0.029694	0.013117
P38570	ITGAE	1.864963314	0.000511	0.011145	0.004923
Q9H8M9	EVA1A	1.853527672	0.004099	0.036122	0.015957
P50454	SERPINH1	1.841899883	0.009367	0.060251	0.026616
Q92626	PXDN	1.823699728	0.000234	0.008378	0.003701
Q96AM1	MRGPRF	1.815895616	0.000522	0.01128	0.004983
Q75N90	FBN3	1.789695284	0.022305	0.101566	0.044867
Q96QV1	HHIP	1.784785087	0.015724	0.085714	0.037864
Q96AY3	FKBP10	1.746275247	4.56E-05	0.003272	0.001445
P55287	CDH11	1.744661516	0.002723	0.028093	0.01241
Q9H4G4	GLIPR2	1.718401007	2.29E-05	0.002269	0.001002
Q76M96	CCDC80	1.702371441	0.018647	0.094074	0.041557
Q9UHI8	ADAMTS1	1.699787042	0.000968	0.016821	0.007431
P14209	CD99	1.697759954	0.000112	0.005154	0.002277
P17302	GJA1	1.648511233	0.000168	0.006747	0.002981
P08133	ANXA6	1.643402913	0.010689	0.066114	0.029206
Q96FE7	PIK3IP1	1.643384549	0.024537	0.107007	0.04727
Q9BRK3	MXRA8	1.618579048	0.00212	0.025351	0.011199
P51911	CNN1	1.612790573	0.007817	0.053353	0.023569
O76061	STC2	1.597366299	0.001524	0.020984	0.00927
Q92519	TRIB2	1.591706981	0.000625	0.012563	0.00555
Q8N7U9	LINC00469	1.589025833	0.005882	0.043738	0.019321

P28300	LOX	1.551627926	0.003361	0.031794	0.014045
P22090	RPS4Y1	1.544324289	0.022417	0.101566	0.044867
Q9HCL0	PCDH18	1.544020533	0.020215	0.097373	0.043014
P08253	MMP2	1.536728534	0.016798	0.089209	0.039408
Q04941	PLP2	1.508794374	0.021295	0.100031	0.044189
Q12884	FAP	1.506132673	0.017037	0.089279	0.039439
P25940	COL5A3	1.502908443	0.013486	0.077976	0.034446
P51790	CLCN3	1.492417897	0.001409	0.020026	0.008847
P08648	ITGA5	1.477465414	0.001924	0.024143	0.010665
Q9Y680	FKBP7	1.460781913	0.013152	0.077086	0.034053
P08758	ANXA5	1.446722304	0.002836	0.02839	0.012541
P12319	FCER1A	1.430128087	3.34E-05	0.002822	0.001247
O95980	RECK	1.429153924	0.006219	0.04598	0.020312
P84157	MXRA7	1.417005988	0.000417	0.010196	0.004504
Q9UKP4	ADAMTS7	1.41031976	3.73E-07	0.000241	0.000107
Q15155	NOMO1	1.398008361	0.007002	0.049979	0.022078
ENST00000379086_NCI-H23_Mis:R399H	P4HA2	1.392434004	0.015949	0.086393	0.038164
Q86SQ3	EMR4P	1.388594955	0.013833	0.079342	0.035049
P17661	DES	1.385243171	0.00437	0.037198	0.016432
P04921	GYPC	1.382399677	3.96E-05	0.002996	0.001323
P55789	GFER	1.373914998	0.001819	0.023397	0.010336
O95450	ADAMTS2	1.364447554	0.024818	0.107684	0.047569

P48509	CD151	1.355960829	0.000708	0.0137	0.006052
P05496	ATP5G1	1.338796173	0.004067	0.03596	0.015885
Q08397	LOXL1	1.327287659	0.019298	0.095672	0.042263
P07093	SERPINE2	1.322615272	0.000923	0.016259	0.007182
P21333	FLNA	1.308257436	0.000261	0.008558	0.003781
P19876	CXCL3	1.286431148	0.000186	0.007349	0.003246
O15427	SLC16A3	1.274249419	0.0165	0.088528	0.039107
P23634	ATP2B4	1.270285797	0.007227	0.050714	0.022403
Q96JY6	PDLIM2	1.262752332	4.08E-06	0.000954	0.000422
Q8IXL7	MSRB3	1.260181225	0.000266	0.008558	0.003781
P13674	P4HA1	1.243864261	0.005635	0.042897	0.01895
P14543	NID1	1.22264481	0.019973	0.097373	0.043014
Q9H9B4	SFXN1	1.219657001	0.012267	0.073651	0.032535
Q15582	TGFBI	1.218613361	0.004519	0.038124	0.016841
Q14108	SCARB2	1.21311293	0.000123	0.00538	0.002377
P15954	COX7C	1.136610105	0.008139	0.054598	0.024119
P03952	KLKB1	1.104252521	0.01657	0.088638	0.039156
Q96N06	SPATA33	1.093524522	0.001033	0.017416	0.007693
P60033	CD81	1.088662494	0.000275	0.008595	0.003797
Q9Y694	SLC22A7	1.083442161	0.004838	0.039591	0.01749
P22692	IGFBP4	1.080248324	0.019059	0.09532	0.042107
Q32P28	LEPRE1	1.076938182	0.002631	0.027955	0.012349

Q9ULI3	HEG1	1.062935688	0.020384	0.097488	0.043065
Q16527	CSRP2	1.040694922	0.01919	0.095506	0.04219
P08670	VIM	1.022111063	0.015006	0.082678	0.036523
P48061	CXCL12	1.015075089	0.00546	0.042062	0.018581
P09429	HMGB1	-1.008233078	3.39E-05	0.002822	0.001247
P18564	ITGB6	-1.026590572	0.000114	0.005154	0.002277
P33261	CYP2C19	-1.028766475	0.01929	0.095672	0.042263
Q13185	CBX3	-1.077389618	3.63E-05	0.002915	0.001288
Q9C0B7	TANGO6	-1.118111193	0.007213	0.050714	0.022403
O95096	NKX2-2	-1.17408716	1.99E-05	0.002229	0.000985
Q8N5P1	ZC3H8	-1.196798342	0.000855	0.015405	0.006805
Q9Y5V0	ZNF706	-1.198453086	0.000333	0.009516	0.004204
Q9H1Z9	TSPAN10	-1.201868581	0.000104	0.005099	0.002253
Q8NEW0	SLC30A7	-1.244369491	0.003099	0.029979	0.013243
P63173	RPL38	-1.245847108	0.00187	0.023938	0.010575
O00555	CACNA1A	-1.249739262	0.000362	0.009989	0.004413
Q32ZL2	LPPR5	-1.285398129	0.000989	0.01696	0.007492
O76075	DFFB	-1.480277449	0.001906	0.024038	0.010619
P61024	CKS1B	-1.659648252	2.69E-05	0.002525	0.001116

Table S4 List of drug hits

The differential drug response hits ranked by number of cell lines they were common in irrespective of the directionality of the response. Differential drug response hits were identified if the difference between the cancer conditioned media (CM) and cancer-associated fibroblast (CAF) CM response (delta) was more than 2 standard deviations away from the mean delta of all drugs at a specific concentration in a cancer cell line. The first 12 drugs that are common in at least 5 cell lines (highlighted above the yellow line) were assessed in the drug validation stage by generating a 11 point IC50 curve for all 9 cancer cell lines. Green=CAF CM sensitivity, pink= CAF CM mediated resistance and grey= CAF CM mediated sensitivity and resistance defined by different concentrations. Chemo=chemotherapy.

		H747	LIM2099	SW620	H1792	H2030	H23	CAPAN1	DANG	MIAPACA2
**Drug**	**Target**	**Colorectal cancer**			**Lung cancer**			**Pancreatic cancer**		
**Ibrutinib (PCI-32765)**	BTK									
**Flutamide**	AR									
**Methotrexate**	Anti-folate/Chemo									
**Zibotentan (ZD4054)**	Endothelin Receptor									
**Venetoclax (ABT-199)**	BCL2									
**Dasatinib (BMS-354825)**	Multi RTK									
**Erdafitinib**	FGFR									
**Erismodegib (NVP-DE225, LDE225)**	Smoothened									
**Galunisertib (LY2157299)**	TGFBR									
**Mercaptopurine (6MP)**	Purine analog/chemo									
**Prednisolone**	Anti-inflammatory/ Chemo									
**Pemetrexed**	Anti-folate/Chemo									
**Vorinostat (SAHA, MK0683)**	HDAC									
**Veliparib (ABT-888)**	PARP									
**AZD4547**	FGFR									
**Bexarotene**	RXR									
**Alpelsib (BYL-719)**	PI3K									
**Cediranib (AZD217)**	VEGFR									
**Fasudil (HA-1077) HCl**	ROCK									
**Fluorouracil (Adrucil)**	Pyrimidine analog/Chemo									
**Pictilisib (GDC-0941)**	PI3K									
**Imatinib (STI571)**	Multi RTK									
**IWR-1-endo**	WNT/β-catenin									
**Niraparib (MK-4827)**	PARP									
**Tamoxifen**	SERM/Chemo									
**SN-38 (7-Ethyl-10-hydroxycamptothecin)**	Topoisomerase I/Chemo									
**17-AAG (KOS953)**	HSP90									
**Crenolanib (CP-868596)**	PDGFR									
**Dabrafenib (GSK2118436)**	BRAF									
**Dacarbazine**	Alkylating agent/Chemo									
**Doxorubicin**	Topoisomerase II/Chemo									
**Entrectinib**	TRK									
**Everolimus (RAD001)**	MTOR									
**Fludarabine**	Purine analog/Chemo									
**HDM201**	HDM2									
**LCL161**	IAPs									
**Ribociclib (LEE011)**	CDK4/6									
**MDV3100 (Enzalutamide)**	AR									
**osimertinib**	EGFR									
**Oxaliplatin**	Alkylating agent/Chemo									
**Topotecan**	Topoisomerase I/Chemo									

		H747	LIM2099	SW620	H1792	H2030	H23	CAPAN1	DANG	MIAPACA2
		Colorectal cancer			Lung cancer			Pancreatic cancer		
**Vincristine sulfate**	Antimicrotubule/ Chemo									
**AG-221 (Enasidenib)**	IDH2									
**Sotorasib (AMG-510)**	KRAS									
**AUY922 (NVP-AUY922)**	HSP90									
**Ceralasertib (AZD6738)**	ATR									
**CCT245737 (SRA737)**	CHK1									
**Decitabine (NSC127716, 5AZA-CdR)**	DNA methylation/ Chemo									
**Encorafenib**	BRAF									
**Epirubicin HCl**	Topoisomerase II/Chemo									
**Gefitinib (ZD1839)**	EGFR									
**Gemcitabine**	Pyrimidine analog/Chemo									
**GSK2636771**	PI3K									
**Lapatinib**	HER2									
**Lenalidomide (CC-5013)**	Immunomodulatory/ Chemo									
**Lenvatinib (E7080)**	VEGFR									
**Panobinostat (LBH589)**	HDAC									
**RO5126766 (CH5126766)**	RAF/MEK									
**Ruxolitinib (INCB018424)**	JAK									
**Sunitinib**	VEGFR									
**Temozolomide**	Alkylating agent/Chemo									
**Trametinib (GSK1120212)**	MEK									
**Vinorelbine**	Antimicrotubule/ Chemo									
**Ivosidenib (AG-120)**	IDH1									
**Selumetinib (AZD6244)**	MEK									
**Empesertib (BAY1161909)**	MPS									
**Elimusertib (BAY1895344)**	ATR									
**Bortezomib (PS-341)**	Proteosome inhibitor/Chemo									
**Alectinib (CH5424802)**	ALK									
**Crizotinib hydrochloride**	ALK									
**Defactinib**	FAK									
**DMXAA (Vadimezan)**	STING/Chemo									
**Ipatasertib (GDC-0068/RG7440)**	AKT									
**Ifosfamide**	Alkylating agent/Chemo									
**Olaparib (AZD2281, Ku-0059436)**	PARP									
**Paclitaxel (Taxol)**	Antimicrotubule/ Chemo									
**Palbociclib (PD 0332991)**	CDK4/6									
**Bemcentinib (R428)**	AXL									
**SCH772984**	ERK									
**Fedratinib (TG101348, SAR302503)**	JAK									

		H747	LIM2099	SW620	H1792	H2030	H23	CAPAN1	DANG	MIAPACA2
		Colorectal cancer			Lung cancer			Pancreatic cancer		
**Thalidomide**	Immunomodulatory/ Chemo									
**Total**		25	25	26	26	29	25	21	29	25
